# Tafenoquine co-administered with dihydroartemisinin–piperaquine for the radical cure of *Plasmodium vivax* malaria (INSPECTOR): a randomised, placebo-controlled, efficacy and safety study

**DOI:** 10.1016/S1473-3099(23)00213-X

**Published:** 2023-10

**Authors:** Inge Sutanto, Amin Soebandrio, Lenny L Ekawati, Krisin Chand, Rintis Noviyanti, Ari Winasti Satyagraha, Decy Subekti, Yulia Widya Santy, Chelzie Crenna-Darusallam, Instiaty Instiaty, Waras Budiman, Catur Bidik Prasetya, Soroy Lardo, Iqbal Elyazar, Stephan Duparc, Eve Cedar, Katie Rolfe, Disala Fernando, Alessandro Berni, Siôn Jones, Jörg-Peter Kleim, Kim Fletcher, Hema Sharma, Ana Martin, Maxine Taylor, Navin Goyal, Justin A Green, Lionel K Tan, J Kevin Baird

**Affiliations:** a Faculty of Medicine, University of Indonesia, Jakarta, Indonesia; b Eijkman Institute for Molecular Biology, Jakarta, Indonesia; c University of Oxford Clinical Research Unit—Indonesia, Jakarta, Indonesia; d Mochtar Riady Institute for Nanotechnology, Banten, Indonesia; e Health Service, Army of the Republic of Indonesia, Jakarta, Indonesia; f Medicines for Malaria Venture, Geneva, Switzerland; g GSK, Brentford, UK; h GSK, Ware, UK; i GSK, Collegeville, PA, USA; j Centre for Tropical Medicine and Global Health, Nuffield Department of Medicine, University of Oxford, Oxford, UK

## Abstract

**Background:**

Tafenoquine, co-administered with chloroquine, is approved for the radical cure (prevention of relapse) of *Plasmodium vivax* malaria. In areas of chloroquine resistance, artemisinin-based combination therapies are used to treat malaria. This study aimed to evaluate tafenoquine plus the artemisinin-based combination therapy dihydroartemisinin–piperaquine for the radical cure of *P vivax* malaria.

**Methods:**

In this double-blind, double-dummy, parallel group study, glucose-6-phosphate dehydrogenase-normal Indonesian soldiers with microscopically confirmed *P vivax* malaria were randomly assigned by means of a computer-generated randomisation schedule (1:1:1) to dihydroartemisinin–piperaquine alone, dihydroartemisinin–piperaquine plus a masked single 300-mg dose of tafenoquine, or dihydroartemisinin–piperaquine plus 14 days of primaquine (15 mg). The primary endpoint was 6-month relapse-free efficacy following tafenoquine plus dihydroartemisinin–piperaquine versus dihydroartemisinin-piperaquine alone in all randomly assigned patients who received at least one dose of masked treatment and had microscopically confirmed *P vivax* at baseline (microbiological intention-to-treat population). Safety was a secondary outcome and the safety population comprised all patients who received at least one dose of masked medication. This study is registered with ClinicalTrials.gov, NCT02802501 and is completed.

**Findings:**

Between April 8, 2018, and Feb 4, 2019, of 164 patients screened for eligibility, 150 were randomly assigned (50 per treatment group). 6-month Kaplan-Meier relapse-free efficacy (microbiological intention to treat) was 11% (95% CI 4–22) in patients treated with dihydroartemisinin–piperaquine alone versus 21% (11–34) in patients treated with tafenoquine plus dihydroartemisinin–piperaquine (hazard ratio 0·44; 95% CI [0·29–0·69]) and 52% (37–65) in the primaquine plus dihydroartemisinin-piperaquine group. Adverse events over the first 28 days were reported in 27 (54%) of 50 patients treated with dihydroartemisinin–piperaquine alone, 29 (58%) of 50 patients treated with tafenoquine plus dihydroartemisinin–piperaquine, and 22 (44%) of 50 patients treated with primaquine plus dihydroartemisinin–piperaquine. Serious adverse events were reported in one (2%) of 50, two (4%) of 50, and two (4%) of 50 of patients, respectively.

**Interpretation:**

Although tafenoquine plus dihydroartemisinin–piperaquine was statistically superior to dihydroartemisinin–piperaquine alone for the radical cure of *P vivax* malaria, the benefit was not clinically meaningful. This contrasts with previous studies in which tafenoquine plus chloroquine was clinically superior to chloroquine alone for radical cure of *P vivax* malaria.

**Funding:**

ExxonMobil, Bill & Melinda Gates Foundation, Newcrest Mining, UK Government all through Medicines for Malaria Venture; and GSK.

**Translation:**

For the Indonesian translation of the abstract see Supplementary Materials section.

## Introduction

*Plasmodium vivax* malaria imposes a substantial global health burden, with more than 3 billion people at risk of infection.^1^ Relapsing malaria caused by activation of dormant hepatic hypnozoites causes greater than 80% of acute attacks in endemic zones resulting in substantial morbidity and onward transmission.^2^

In many endemic countries, “radical cure” of *P vivax* consists of combining a blood schizontocide, chloroquine, with a liver stage hypnozoitocide, primaquine, to prevent relapse from hepatic latency. WHO recommends a 7-day or 14-day course of primaquine;^3^ however, in clinical practice this regimen is often unsupervised, resulting in poor adherence and compromised effectiveness, especially of the 14-day course.^4^ Tafenoquine (Kozenis–Krintafel, GSK, London, UK) is a single-dose, long-acting synthetic analogue of primaquine that facilitates treatment adherence and, when co-administered with chloroquine, can reduce the risk of recurrence of *P vivax* malaria by 70% compared with chloroquine alone.^5^, ^6^

**Research in context**

**Evidence before this study**

The efficacy of a single 300-mg dose of tafenoquine co-administered with chloroquine for the radical cure of *Plasmodium vivax* malaria has been previously established in randomised controlled studies. In the phase 3 DETECTIVE study done in Ethiopia, Peru, Brazil, Cambodia, Thailand, and the Philippines, recurrence-free efficacy rates at 6 months (modified intention-to-treat population) were 62·4% (95% CI 54·9–69·0) in the tafenoquine group, 27·7% (19·6–36·6) in the placebo group, and 69·6% (60·2–77·1) in the primaquine group. In a patient-level meta-analysis of the phase 3 DETECTIVE study and the phase 3 GATHER study (done in Peru, Brazil, Colombia, Viet Nam, and Thailand; per-protocol population), recurrence-free efficacy rates at 6 months were 67·0% (61·0–72·3) in the tafenoquine group and 72·8% (65·6–78·8) in the primaquine group. Dihydroartemisinin–piperaquine is an artemisinin-based combination therapy used as an alternative to chloroquine for treatment of malaria. A PubMed search for articles published from database inception to July 1, 2022 containing the terms “vivax malaria”, “tafenoquine”, “dihydroartemisinin” or “artenimol”, and “piperaquine”, with no language restrictions, revealed only a single article of relevance: a drug–drug interaction study in healthy volunteers investigating the pharmacokinetic interaction between tafenoquine and the artemisinin-based combination therapies, dihydroartemisinin-piperaquine, and artemether-lumefantrine. There were no reported studies evaluating the efficacy and safety of tafenoquine co-administered with artemisinin-based combination therapies for the radical cure of *P vivax* malaria.

**Added value of this study**

This is the first clinical study to evaluate the efficacy and safety of a single 300-mg dose of tafenoquine co-administered with dihydroartemisinin–piperaquine for the radical cure of *P vivax* malaria. No clinically meaningful benefit was observed. The benefit of primaquine (15 mg) plus dihydroartemisinin–piperaquine was clinically meaningful, although nearly half of all patients relapsed within 6 months of treatment.

**Implications of all the available evidence**

This study does not support co-administration of a single 300-mg dose tafenoquine with dihydroartemisinin–piperaquine for the radical cure of *P vivax* malaria. These results are important for national malaria control programmes in countries which might consider using artemisinin-based combination therapies for *P vivax* malaria.

The emergence of chloroquine resistance^7^ has meant that some endemic countries have adopted artemisinin-based combination therapies for malaria treatment. In Indonesia, where approximately 500 000 clinical cases of *P vivax* malaria were reported in 2019,^8^ chloroquine resistance is widespread^9^, ^10^ and local guidelines advocate dihydroartemisinin–piperaquine for 3 days plus a 14-day course of primaquine (0·25 mg/kg or 15 mg daily) for radical cure of *P vivax* malaria.^11^ Dihydroartemisinin–piperaquine has proven efficacy against blood stage *P vivax* infection in regions in which chloroquine resistance is common^12^ and, when co-administered with primaquine 15 mg for 14 days, has previously achieved recurrence-free efficacy rates of over 90% in North Sumatra.^13^

The INSPECTOR study (Indonesian Study Proving Efficacy of Combination Therapy on Relapse) is the first study to evaluate co-administration of tafenoquine with dihydroartemisinin–piperaquine for the radical cure of *P vivax* malaria. The study was designed to show superiority of a single 300-mg dose of tafenoquine co-administered with standard doses of dihydroartemisinin–piperaquine, compared with dihydroartemisinin–piperaquine alone, for prevention of relapse of *P vivax* malaria over 6 months in Indonesian soldiers returning to malaria-free military bases in Malang and Madiun following deployment to an area with high *P vivax* endemicity who were phenotypically glucose-6-phosphate dehydrogenase-normal (appendix 2 p 21).^14^

## Methods

### Study design and participants

This double-blind, double-dummy, randomised, parallel group study enrolled soldiers from two battalions based in East Java (battalion 1 at Malang; battalion 2 at Madiun; appendix 2 p 21). Following a 9-month deployment to the same region of Indonesian Papua, the battalions returned to East Java in immediate sequence. After 2 weeks of leave, soldiers returned to their bases and were advised about the study, with an emphasis on the importance of providing voluntary informed consent if they wanted to take part. Soldiers subsequently testing positive for *P vivax* malaria by microscopy at their army bases were invited to participate in the study. A clinic, laboratory, and pharmacy were established at both bases for the duration of the study.

Patients were eligible for enrolment if they were male, aged at least 18 years, had microscopically confirmed *P vivax* malaria with asexual parasite density greater than 20/μL (or mixed infection with *Plasmodium falciparum*), and were glucose-6-phosphate dehydrogenase-normal as assessed by the qualitative fluorescent spot test (Trinity Biotech, Bray, Ireland), as primaquine and tafenoquine can cause acute haemolytic anaemia in patients with glucose-6-phosphate dehydrogenase deficiency. Glucose-6-phosphate dehydrogenase status was confirmed shortly after randomisation by quantitative spectrophotometric assay (Trinity Biotech, Bray, Ireland or Pointe Scientific, Canton, MI, USA).

The main exclusion criteria were severe *P vivax* malaria (as defined by WHO), severe vomiting, corrected QT interval (QTc) of at least 450 msec, screening haemoglobin concentration less than 8 g/dL, alanine aminotransferase greater than twice the upper limit of normal, consumption of antimalarial drugs or drugs known to prolong the QTc interval in the past 30 days, or any other contraindication to administration of dihydroartemisinin–piperaquine or primaquine. A complete list of exclusion criteria is included in the appendix 2 (pp 3–5).

The trial was designed to support the use of tafenoquine 300 mg with dihydroartemisinin–piperaquine. The underlying relapse rate following a course of dihydroartemisinin–piperaquine cannot be assumed from historical data owing to the natural variation in both infection rates and relapse rates among infected subjects. The dihydroartemisinin–piperaquine alone group was included as a relapse-prevention placebo control to provide an efficacy benchmark in the same setting and at the same time as the study treatment groups. The inclusion of the primaquine group provided a context against which to interpret the observed tafenoquine efficacy rate. A statistical comparison for non-inferiority between tafenoquine and primaquine was not done; such a comparison would require a considerably larger sample size and was not the main objective of the study, which was to estimate the efficacy of tafenoquine.

The study complied with Good Clinical Practice, the Declaration of Helsinki, and relevant regulatory requirements. Ethics approval was obtained from the Oxford Tropical Research Ethics Committee (Project 9-16) and the Faculty of Medicine Universitas Indonesia Ethics Committee (FKUI 593/UNs.F1/ETIK/2016 dated July 18, 2016). Written informed consent was obtained individually from study patients and affirmed by an independent peer witness. Army rank superiors were not permitted to witness or participate in the consenting process to avoid any perception of coercion. The protocol was amended once on April 20, 2017 (before study start) to include an additional secondary objective requested by the Indonesian regulatory agency: comparison of dihydroartemisinin–piperaquine plus primaquine (as per national treatment guidelines) with dihydroartemisinin–piperaquine alone. The full protocol is available online (NCT 02802501).

### Randomisation and masking

Eligible patients were randomly assigned in a 1:1:1 ratio to dihydroartemisinin–piperaquine alone, dihydroartemisinin–piperaquine plus tafenoquine, or dihydroartemisinin–piperaquine plus primaquine. All patients received open-label dihydroartemisinin–piperaquine for 3 days plus masked treatment as follows: tafenoquine 300-mg single dose on day 1 and placebo for primaquine on days 1–14; primaquine 15 mg on days 1–14 and placebo for tafenoquine on day 1; or placebo for tafenoquine on day 1 and placebo for primaquine on days 1–14. The study sponsor provided a computer-generated randomisation schedule to the site. Visually matched tafenoquine and primaquine placebos were used to maintain masking of site staff, patients, and sponsor personnel.

As methaemoglobin increases are associated with use of 8-aminoquinolines such as primaquine and tafenoquine, methaemoglobin assessments were done by an independent site assessor to avoid unmasking. Additionally, independent sponsor staff processed the methaemoglobin data. The independent methaemoglobin site and sponsor staff did not have access to other study data.

### Procedures

Study treatments were supplied by the sponsor as eurartesim tablets containing dihydroartemisinin 40 mg and piperaquine 320 mg (Alfasigma, Bologna, Italy), tafenoquine 150-mg tablets (GSK, London, UK), and primaquine formulated as over-encapsulated 15-mg tablets (Sanofi-Aventis, Bridgewater, NJ, USA). All patients received open-label, oral dihydroartemisinin–piperaquine daily over 3 consecutive days (days 1–3), according to weight (three tablets <75 kg or four tablets ≥75 kg). Tafenoquine (or matched placebo) was given as a single oral 300-mg dose (two × 150-mg tablets) on either day 1 or day 2. Primaquine (or matched primaquine placebo) was administered as a single oral 15-mg daily dose for 14 consecutive days starting on day 1 or day 2. All study medications were administered under direct supervision by study staff in the clinic.

Following informed consent, screening assessments were done and all patients who were eligible to receive dihydroartemisinin–piperaquine on the basis of labelling received their first dose of dihydroartemisinin–piperaquine at least 3 h after their last meal. Once laboratory results confirmed continued eligibility, patients were randomly assigned to masked study medication, with the first dose given, with food, at least 3 h after dihydroartemisinin–piperaquine, on day 1 or day 2. Blood smears for parasite assessment were done twice daily until two consecutive negative smears were obtained. After completion of the dosing period, patients attended a further seven follow-up visits (on days 21, 28, 60, 90, 120, 150, and 180).

Patients were evaluated by clinical assessments and laboratory investigations including Giemsa-stained blood smears for parasitology, 12-lead electrocardiograms, haematology and clinical chemistry tests, and methaemoglobin measurement by means of a non-invasive pulse oximeter (Masimo, Irvine, CA, USA) at screening and selected visits throughout the study. After their initial in-patient treatments, patients were instructed to promptly return to the clinic (open 24 h) if they had malaria symptoms. Patients who returned with *P vivax* positive blood smears with or without symptoms were treated with rescue medication: dihydroartemisinin–piperaquine plus open-label primaquine 0·5 mg/kg daily for 14 days (see appendix 2 p 22 for study design).

Blood samples were taken for tafenoquine pharmacokinetic analysis, cytochrome P-450 2D6 (CYP2D6) genotyping and parasite microsatellite DNA analysis (to establish the proportion of heterologous and homologous relapses). Relapse was defined as genetically homologous when *P vivax* clones were identical to baseline in all markers. Masked external quality assurance was done on 20% of samples for CYP2D6 analysis, 20 paired samples for parasite DNA analysis, all positive blood films, and 10% of negative blood films (further details of methods are provided in the appendix 2 p 6).

All patients who were relapse free at 6 months received high-dose open-label primaquine (ie, 0·5 mg/kg daily) for 14 days at the end of the study to minimise the likelihood of relapse.

### Outcomes

The primary outcome was relapse-free efficacy over 6 months, defined as clearance of initial infection without subsequent microscopically confirmed recurrence or receipt of other antimalarial treatment. Secondary outcomes were relapse-free efficacy over 4 months, time to fever clearance, time to parasite clearance, and percentage of patients with recrudescence (genetically homologous recurrence within 14 days) to ensure the blood stage efficacy of dihydroartemisinin–piperaquine. Safety assessments included frequency and severity of adverse events and review of 12-lead electrocardiograms, vital signs, and laboratory values. Tafenoquine pharmacokinetic assessments were also planned.

The incidence of genetically homologous infections was established by five microsatellite markers. Additionally, the influence of human CYP2D6 polymorphisms on relapse was explored post-hoc by means of a CYP2D6 activity score system (see appendix 2 p 6).

### Statistical methods

The primary comparison of interest was relapse-free efficacy over 6 months for tafenoquine plus dihydroartemisinin–piperaquine versus dihydroartemisinin–piperaquine alone. By means of the log-rank test for the primary comparison to detect a clinically meaningful difference of 35% in relapse-free survival rates over 6 months, and assuming a 50% rate on dihydroartemisinin–piperaquine alone,^15^ a sample size of 50 patients per group (150 patients in total) was required to provide at least 90% power, assuming 10% of patients withdrew.

All randomly assigned patients who received at least one dose of masked treatment and had microscopically confirmed *P vivax* at baseline were included in the primary and secondary efficacy analyses (microbiological intention-to-treat population). The per-protocol population included all patients in the microbiological intention-to-treat population for whom there were no major protocol violations. The safety population comprised all patients who received at least one dose of masked medication. The primary treatment comparison in the study was tafenoquine plus dihydroartemisinin–piperaquine versus dihydroartemisinin–piperaquine alone. Although other treatment comparisons were made (primaquine plus dihydroartemisinin–piperaquine *vs* dihydroartemisinin–piperaquine alone, and tafenoquine plus dihydroartemisinin–piperaquine versus primaquine plus dihydroartemisinin–piperaquine), these were considered secondary and hence no adjustments for multiplicity were made.

The primary endpoint, time to relapse over 6 months, was summarised by means of Kaplan-Meier estimates and analysed by means of a Cox's proportional-hazards model, adjusting for battalion, for the microbiological intention-to-treat (primary) and per-protocol (sensitivity) populations. Of note, the protocol described a log-rank test, but this was amended in the study analysis plan (finalised before the unmasking of the study) to Cox's proportional hazards. Patients were censored if they did not show initial clearance of *P vivax* parasitaemia, took an antimalarial drug post-baseline without confirmed *P vivax,* or did not have a 6-month assessment within the defined time window. Further sensitivity analyses were done (microbiological intention-to-treat population), which analysed the proportion of participants who were relapse free at 4 and 6 months by means of logistic regression analyses (adjusting for battalion, post-hoc analysis). Treatment by covariate interaction (at 10% significance level) was assessed by means of Cox's proportional-hazards model to evaluate the effect of battalion (ie, battalion 1 or 2), baseline *P vivax* parasite count and patient weight on the treatment effect. No adjustments for other covariates were made. Clearance times for *P vivax* and fever were estimated by means of Kaplan-Meier methods. The effect of CYP2D6 activity score or metaboliser class on relapse within each treatment group was investigated by logistic regression (post-hoc analysis). Other efficacy and safety endpoints were summarised by means of descriptive statistics. Statistical analyses were done by means of the SAS software, version 9.4. Masked safety data were reviewed on a monthly basis by a GSK–MMV safety review board which included a reviewer independent of the study sponsors. This study is registered with ClinicalTrials.gov, NCT02802501.

### Role of the funding source

The study sponsor (GSK) was responsible for study monitoring, data management, data analysis, and writing of the clinical report.

## Results

**Figure 1:**
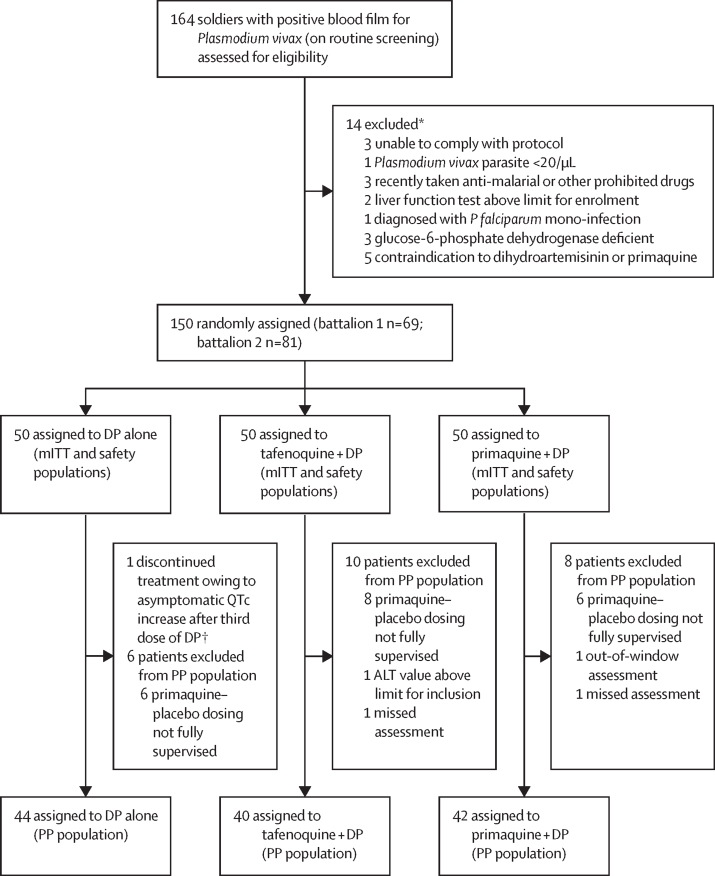
Trial profile ALT=alanine aminotransferase. DP=dihydroartemisinin–piperaquine. mITT=microbiological intention-to-treat. PP=per-protocol. PQ=primaquine. *Participants can be excluded for more than one reason. †The patient discontinued treatment, but continued and completed the study. The safety population included all patients who were randomly assigned and received at least one dose of masked study medication. The mITT population was a subgroup of the safety population who had microscopically confirmed *Plasmodium vivax* parasitaemia at baseline. The per-protocol population was a subgroup of patients in the mITT population who had no major protocol violations.

Between April 8, 2018, and Feb 4, 2019, of 164 patients screened for eligibility, 150 were randomly assigned to masked study treatment; 50 per treatment group (battalion 1 n=69; battalion 2 n=81; figure 1). Local epidemiology data for East Java indicated no local malaria transmission at the study bases during the study period, thus eliminating the possibility of reinfection.^16^ All patients were included in both safety and microbiological intention-to-treat populations and completed the study. Baseline characteristics were similar across treatment groups (table 1) and between battalions (appendix 2 p 7). With the exception of one patient in the dihydroartemisinin–piperaquine alone group who discontinued primaquine placebo tablets on day 3 following a serious adverse event, all patients completed masked treatment. A total of 24 patients (16%) were excluded from the per-protocol population (six patients on dihydroartemisinin–piperaquine alone, ten patients on tafenoquine plus dihydroartemisinin–piperaquine, and eight patients on primaquine plus dihydroartemisinin–piperaquine), primarily because of patients not receiving fully supervised primaquine or primaquine placebo medication as a result of unforeseen off-base assignments during the dosing period (n=20). Other reasons for exclusion included three patients who missed, or were outside, the required window for visits or assessments, and one patient with baseline alanine aminotransferase of more than twice the upper limit of normal.

**Table 1:** Baseline characteristics for safety population

		**Dihydroartemisinin–piperaquine alone group (n=50)**	**Tafenoquine plus dihydroartemisinin–piperaquine group (n=50)**	**Primaquine plus dihydroartemisinin–piperaquine group (n=50)**
Age, years		28·3 (4·3)	29·4 (5·1)	28·6 (5·6)
Weight, kg		70·9 (9·9)	69·7 (10·9)	70·4 (8·9)
Height, cm		169·4 (4·0)	170·2 (3·5)	169·3 (3·8)
BMI, kg/m^2^		24·7 (3·6)	24·1 (3·8)	24·5 (2·6)
Previous episode(s) of malaria		48 (96)	46 (92)	49 (98)
Glucose-6-phosphate dehydrogenase enzyme activity (IU/g Hb), median (range)*†		7·5 (5·8–12·8)	7·5 (5·8–10·8)	7·6 (6·1–11·9)
Cytochrome P-450 2D6 metaboliser class‡				
	Poor	1 (2%)	0	0
	Intermediate	23 (46%)	23 (46%)	20 (40%)
	Normal	25 (50%)	27 (54%)	29 (58%)
	Ultra	1 (2%)	0	1 (2%)
Co-infection with *P falciparum*		0	0	0

Data are mean (SD), n (%), and median (range). IU=international units.

* Glucose-6-phosphate dehydrogenase activity ≥5·1 IU/gHb was considered normal based on ≥70% of the site median 7·29 (range 5·77–11·92) IU/gHb established from the first 33 recruited patients who were G6PD normal by the fluorescent spot test.

† There were no discordant G6PD results between the qualitative fluorescent spot test and the quantitative spectrophotometric assay in any randomly assigned patient.

‡ Poor activity score 0; intermediate=activity score=0·25 to 1; normal activity score=1·25–2·25; ultra activity score >2·25 (see appendix p 6).

**Figure 2:**
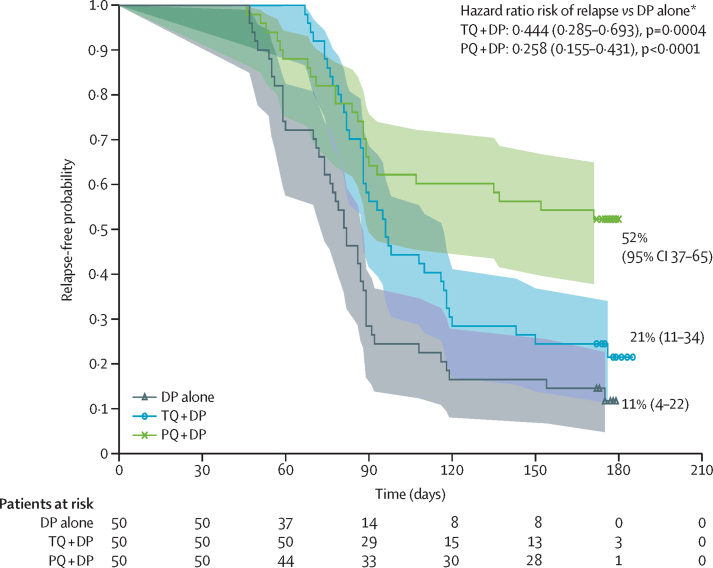
Kaplan-Meier survival curves for 6-month relapse-free efficacy for the microbiological intention-to-treat population DP=dihydroartemisinin–piperaquine. PQ=primaquine. TQ=tafenoquine. *Estimated from Cox's proportional-hazards analysis adjusting for battalion. A hazard ratio of <1 indicates a lower chance of relapse compared with the reference treatment. No patients met the criteria for censoring.

In the microbiological intention-to-treat population, 44 patients (88%) in the dihydroartemisinin–piperaquine alone group, 39 patients (78%) in the tafenoquine plus dihydroartemisinin–piperaquine group and 24 patients (48%) in the primaquine plus dihydroartemisinin–piperaquine group had microscopically confirmed *P vivax* relapse during the 6-month follow-up period. Kaplan-Meier estimates of relapse-free efficacy over 6 months were 11·2% (95% CI 4·2–22·1) for dihydroartemisinin–piperaquine alone, 21·0% (10·7–33·6) for tafenoquine plus dihydroartemisinin–piperaquine, and 52·0% (37·4–64·7) for primaquine plus dihydroartemisinin–piperaquine. Tafenoquine plus dihydroartemisinin–piperaquine reduced the risk of relapse at any time by 55·6% versus dihydroartemisinin–piperaquine alone (hazard ratio [HR] 0·44; 95% CI 0·29–0·69; p=0·0004; figure 2). In the logistic regression analysis, the odds of being relapse-free at 6 months following tafenoquine plus dihydroartemisinin–piperaquine was not significantly different from dihydroartemisinin–piperaquine alone (table 2). Results for the per-protocol population were consistent with results for the microbiological intention-to-treat population (appendix 2 p 9). The patients' battalion was found to have a significant effect on the treatment effect, whereas neither patient weight nor baseline asexual parasite count significantly influenced the results (appendix 2 pp 10–11).

**Table 2:** Logistic regression analysis of relapse-free efficacy at 6 months (modified intention to treat population)

	**n**	**Patients relapse-free**	**Patients relapsed**	**Comparison with DP alone**			**Comparison with primaquine plus DP**	
				Adjusted odds ratio of relapse*	95% CI	p value	Adjusted odds ratio of relapse†	95% CI
DP alone	50	6 (12%)	44 (88%)	..	..	..	..	..
Tafenoquine plus DP	50	11 (22%)	39 (78%)	0·43	0·14–1·35	0·149	4·57	1·75–11·97
Primaquineplus DP	50	26 (52%)	24 (48%)	0·09	0·03–0·29	<0·0001	..	..

Data are n (%) unless stated otherwise. DP=dihydroartemisinin-piperaquine. Model includes terms for battalion and treatment. Patients who did not show initial clearance of *Plasmodium vivax* parasitemia, take a concomitant medication with antimalarial activity, have a missing day 180 assessment, or have a zero *Plasmodium vivax* asexual parasite count at baseline were to be excluded from the analysis. No patients met these criteria.

* An odds ratio <1 represents a smaller chance of relapse compared with DP alone.

† An odds ratio <1 represents a smaller chance of relapse compared with primaquine plus DP.

Primaquine plus dihydroartemisinin–piperaquine reduced the risk of relapse over 6 months by 74·2% versus dihydroartemisinin–piperaquine alone (HR 0·26; 95% CI 0·16–0·43; figure 2). In the logistic regression analysis, the odds of being relapse free at 6 months was also higher following primaquine plus dihydroartemisinin–piperaquine versus dihydroartemisinin–piperaquine alone (table 2). As there was a violation of the proportional hazards' assumption, the comparison of tafenoquine plus dihydroartemisinin–piperaquine versus primaquine plus dihydroartemisinin–piperaquine, HR is considered unreliable. The odds ratio (OR) is therefore used for comparison, with the odds of relapsing following tafenoquine plus dihydroartemisinin–piperaquine higher than primaquine plus dihydroartemisinin–piperaquine (OR 4·57; 95% CI 1·75–11·97).

The percentage of relapse-free patients in all treatment groups over 6 months was higher in battalion 1 than battalion 2 (battalion 1 dihydroartemisinin–piperaquine alone five [22%] of 23; tafenoquine plus dihydroartemisinin–piperaquine seven [32%] of 22; primaquine plus dihydroartemisinin–piperaquine 19 [79%] of 24; battalion 2 dihydroartemisinin–piperaquine alone one [4%] of 27; tafenoquine plus dihydroartemisinin–piperaquine four [14%] of 28; primaquine plus dihydroartemisinin–piperaquine seven [27%] of 26). The reduction in the 6-month relapse risk for tafenoquine plus dihydroartemisinin–piperaquine versus dihydroartemisinin–piperaquine alone was greater for battalion 2 (battalion 1 HR 0·70; 95% CI 0·35–1·39; battalion 2: HR 0·38; 0·21–0·67; see appendix 2 pp 12, 23).

Relapse-free efficacy trends over 4 months were similar to the 6-month results (appendix 2 p 13). Clearance of parasitaemia was achieved in all patients (both asexual and gametocytes) within the first 3 study days and the times to parasite clearance and to fever clearance were similar across treatment groups (appendix 2 p 14). No patients had recrudescence, defined as a genetically homologous relapse, within the first 14 days of the study.

Treatment groups were well matched for CYP2D6 metaboliser class with 67 (45%) of 150 being categorised as poor or intermediate metabolisers (categorised post-hoc according to Clinical Pharmacogenetics Implementation Consortium guidelines). Logistic regression analyses showed no significant effect of CYP2D6 activity score (appendix 2 p 15) or metaboliser class on 6-month relapse-free efficacy in any treatment group (appendix 2 p 16).

Overall, 88 (83%) of 106 of all first relapses were genetically heterologous by microsatellite genotyping and the proportion of heterologous to homologous relapses was not influenced by treatment (appendix 2 p 24).

The adverse event profile was similar across treatment groups, although the proportion of patients in the primaquine plus dihydroartemisinin–piperaquine group reporting any adverse event was lower than for other groups (table 3; appendix 2 pp 17, 25). Most adverse events were rated mild to moderate (≥94% in each treatment group). No patients withdrew from the study owing to an adverse event. Predefined adverse events of special interest are summarised in the appendix 2 (p 19).

**Table 3:** Most frequent adverse events and serious adverse events during the double-blind treatment phase (safety population)

	**DP alone (n=50)**	**Tafenoquine plus DP (n=50)**	**Primaquine plus DP (n=50)**
Most frequent adverse events occurring in ≥5% in any treatment group up to day 29*			
Any event	27 (54%)	29 (58%)	22 (44%)
Upper respiratory tract infection	6 (12%)	6 (12%)	4 (8%)
Electrocardiogram QT prolonged	4 (8%)	6 (12%)	2 (4%)
Nasopharyngitis	3 (6%)	4 (8%)	1 (2%)
Vomiting	1 (2%)	3 (6%)	0
Chills	0	3 (6%)	0
Headache	2 (4%)	2 (4%)	3 (6%)
Dyspepsia	1 (2%)	1 (2%)	3 (6%)
Asthenia	3 (6%)	1 (2%)	1 (2%)
**Serious adverse events**			
Any event	1 (2%)	2 (4%)	2 (4%)
Cholelithiasis	0	0	1 (2%)
Drug-induced liver injury	0	1† (2%)	0
Hand fracture	0	1 (2%)	0
Electrocardiogram QT prolonged	1 (2%)	0	0
Hypertensive urgency	0	0	1 (2%)

Data are n (%). DP=dihydroartemisinin–piperaquine.

* Only adverse events with an onset up to day 29 are presented. The interpretation of the incidence of adverse events after day 29 is likely to be confounded by medication administered to treat relapses (primaquine plus DP).

† One patient was diagnosed with grade 1 drug-induced liver injury at the time of their first *Plasmodium vivax* relapse, 84 days after receiving tafenoquine plus DP.

Five serious adverse events were reported during the 6-month follow-up of which two were considered possibly related to treatment by the investigator (table 3). One patient in the dihydroartemisinin–piperaquine alone group discontinued masked treatment owing to a transient, asymptomatic increase in QT corrected by means of Fredericia's formula (QTcF) from a baseline value of 438·2 ms to 510·2 ms after the third daily dose of dihydroartemisinin–piperaquine, but remained in the study. One patient with a history of hypertension in the primaquine plus dihydroartemisinin–piperaquine group had severe hypertensive urgency (peak blood pressure 210/130 mm Hg on day 10), but with no evidence of target organ damage, and which resolved following antihypertensive treatment. In addition, one patient in the tafenoquine plus dihydroartemisinin–piperaquine group had mild, asymptomatic increases in alanine aminotransferase (>3 × upper limit of normal) and total bilirubin (>2 × upper limit of normal), indicating possible drug-induced liver injury, following relapse of malaria 84 days after tafenoquine administration, and approximately 5 h after the first dose of dihydroartemisinin–piperaquine plus primaquine (30 mg) for relapse treatment. The event resolved within 7 days and was thought by the investigator to be more likely to be related to relapse of malaria itself or the acute relapse medication, rather than to tafenoquine.

A higher percentage of patients in the tafenoquine plus dihydroartemisinin–piperaquine group (12 [24%] of 50) had a clinically significant QTcF prolongation (defined as ≥60 msec change from baseline) on the 12-lead electrocardiogram (dihydroartemisinin–piperaquine alone group seven [14%] of 50; primaquine plus dihydroartemisinin–piperaquine group five [10%] of 50). In all cases, QTcF prolongation was asymptomatic and returned to normal within 1 week without intervention (appendix 2 p 20). Only one patient, in the dihydroartemisinin–piperaquine alone group, had a QTcF value of more than 500 msec (described previously).

**Figure 3:**
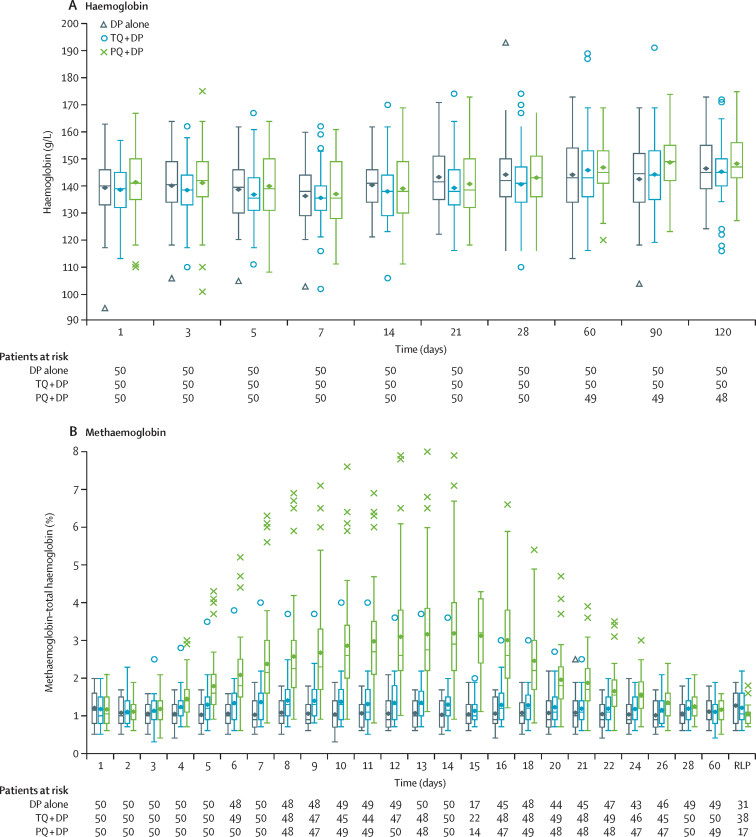
Box plot of haemoglobin and methaemoglobin values over time (safety population) DP=dihydroartemisinin-piperaquine. PQ=primaquine. RLP=relapse visit. TQ=tafenoquine.

Haemoglobin values were similar between treatment groups (figure 3A) and no patient had a protocol-defined haemolytic serious adverse event (decrease of ≥30% or >3·0 g/dL from baseline or a drop in absolute haemoglobin to below 7·0 g/dL in the first 15 days). Asymptomatic increases in methaemoglobin were observed over the first 2 weeks, returning to baseline concentrations by day 28. The greatest methaemoglobin increase was in the primaquine plus dihydroartemisinin–piperaquine group (median [range] 2·9% [0·9–7·9]) versus tafenoquine plus dihydroartemisinin–piperaquine (1·3% [0·7–3·7]) and dihydroartemisinin–piperaquine alone (1·0% [0·5–1·8]; figure 3B). Apart from a small increase in serum creatinine in the tafenoquine plus dihydroartemisinin–piperaquine group (maximum increase 3·22 ± 10·89 μmol/L on day 14), there were no notable clinical chemistry findings.

Owing to various logistical factors including the global response to the COVID-19 pandemic, it has not, to date, been possible to transport samples for pharmacokinetic analyses to a validated laboratory outside of Indonesia. Pharmacokinetic data from study patients cannot therefore be included in this publication.

## Discussion

This is the first study to evaluate the efficacy and safety of a single 300-mg dose of tafenoquine co-administered with an artemisinin-based combination therapy for the radical cure of *P vivax* malaria. A dihydroartemisinin–piperaquine alone group provided a placebo control for the primary assessment of tafenoquine plus dihydroartemisinin–piperaquine efficacy for relapse prevention. Inclusion of a placebo control was considered justifiable as all patients received dihydroartemisinin–piperaquine for the acute illness and were closely monitored for relapse on an army base, thereby ensuring that any episode of recurrent malaria could be treated promptly. Tafenoquine plus dihydroartemisinin–piperaquine reduced the risk of relapse versus dihydroartemisinin–piperaquine alone (21% *vs* 11%, respectively). However, a 10% difference in relapse-free efficacy was not considered clinically meaningful. The efficacy of tafenoquine plus dihydroartemisinin–piperaquine was lower than observed in previous studies of tafenoquine co-administered with chloroquine.^5^ Tafenoquine plus dihydroartemisinin–piperaquine was less efficacious than primaquine plus dihydroartemisinin–piperaquine, with the odds of relapse over 6 months more than four times higher.

The reasons for the lack of clinically relevant efficacy with tafenoquine plus dihydroartemisinin–piperaquine are unknown. Although no pharmacokinetic data are yet available, a pharmacokinetic interaction is considered unlikely given that there was no clinically significant pharmacokinetic interaction between tafenoquine and dihydroartemisinin–piperaquine in a previous healthy volunteer study.^17^ Any future availability of pharmacokinetic data from study patients is not expected to change the interpretation of the findings from this study. Although patients could receive tafenoquine on either day 1 or day 2, because dihydroartemisinin–piperaquine was administered on day 1, day 2, and day 3, and dihydroartemisinin is rapidly eliminated from the bloodstream, the day of administration of tafenoquine is not expected to affect therapeutic efficacy. Furthermore, non-clinical data have shown no effect on tafenoquine or dihydroartemisinin pharmacokinetics when these medications are co-administered (Gamo F J, GSK, personal communication). Further non-clinical work is still ongoing and should provide additional insights into this research area.

Previous studies of primaquine plus chloroquine indicate that diminished primaquine efficacy might be associated with impaired CYP2D6 activity.^18^ In contrast, clinical trial data suggest that tafenoquine efficacy is not affected by CYP2D6 status, but differentiation between relapse and reinfection was not possible in these studies.^5^, ^6^ Although approximately half the patients in this study were genotypically categorised as having impaired CYP2D6 metaboliser status, no influence of CYP2D6 metaboliser status on efficacy of tafenoquine or primaquine was observed. However, this trial was not powered to detect possible effects of impaired CYP2D6 activity on therapeutic efficacy, and such effects might have been obscured by dominating treatment failures owing to other causes.

The soldiers' battalion had a significant effect on estimates of efficacy, whereas other covariates (weight and baseline parasite counts) exerted no significant influence on the primary endpoint. Although these observations did not change the overall interpretation of the study (treatment differences were in the same direction in both battalions), there was a notably higher relapse rate across all treatment groups in the second battalion compared with the first battalion, which could not be explained by differences in study conduct or drug supplies. Despite both battalions having similar baseline characteristics and being deployed to the same region of Indonesian Papua for similar durations, sharp differences in transmission intensity within that region are believed to have occurred. Battalion 1 had been working in a sparsely populated and forested sector, whereas battalion 2 had been operating in a relatively settled (cleared forest) and populated area—conditions conducive to relatively light and heavy malaria transmission, respectively—on the basis of the dominant anopheline vectors in New Guinea, which prefer open sunlight to shady forests.^19^ Thus, the differences in efficacy observed might be due to a higher frequency of soldiers having had an absence of hypnozoites at the time of administration of antirelapse therapy in battalion 1 relative to those in battalion 2.

Consideration has been given to whether the 300-mg dose of tafenoquine used in this study might be suboptimal.^20^, ^21^ However, in light of both an extensive database of tafenoquine clinical studies and the dose-dependent haemolytic risk associated with 8-aminoquinolines, the tafenoquine 300-mg dose is considered to provide the optimal balance of benefit versus risk. Evaluation of tafenoquine 600 mg led to a minimal efficacy increase over tafenoquine 300 mg in the phase 2b dose-ranging study when co-administered with chloroquine in geographical areas other than Indonesian Papua (known for its high *P vivax* burden); relapse-free efficacy at 6 months was 91·9% (tafenoquine 600 mg plus chloroquine) versus 89·2% (tafenoquine 300 mg plus chloroquine).^15^ Population pharmacokinetic modelling also provided support for tafenoquine 300 mg,^22^ and this modelling was validated in paediatric patients.^23^ A more likely explanation than dose comes from evidence showing potentiation of primaquine activity when chloroquine is given as partner blood schizontocide in radical cure.^24^, ^25^, ^26^ Results from non-clinical malaria models indicate that a lower tafenoquine dose is effective in preventing relapse when combined with chloroquine compared with the dose of tafenoquine required if given alone.^27^, ^28^ Such potentiation has not been shown with dihydroartemisinin–piperaquine co-administered with tafenoquine. Thus, the most likely determinant of the relatively low efficacy observed in the current trial was the partner blood schizontocide (dihydroartemisinin–piperaquine).

Patients in this study were infected with *P vivax* in Indonesian Papua, a region where the Chesson strain of *P vivax* originated, a type which is characterised by frequently occurring relapses and tolerance to low-dose primaquine.^29^ Two similar studies done in soldiers returning from Indonesian Papua indicate that higher primaquine doses (ie, 0·5 mg/kg for 14 days) are associated with superior efficacy than in the current study, achieving more than 90% relapse-free efficacy rates.^10^, ^30^ Although WHO recommends high-dose primaquine regimens for *P vivax* radical cure in southeast Asia, uptake is low because of the haemolytic risk associated with 8-aminoquinoline treatment in the absence of G6PD testing. Therefore, the current study used low-dose primaquine (15 mg/day for 14 days) in accordance with Indonesian national treatment guidelines. The 6-month relapse-free efficacy with this primaquine regimen was 52% compared with 11% for dihydroartemisinin–piperaquine alone which is lower than in previous studies with primaquine 15 mg/day plus chloroquine done in other countries^5^, ^6^, ^15^ or primaquine 15 mg/day plus an artemisinin-based combination therapy in hypoendemic to mesoendemic North Sumatra, Indonesia.^13^ Degree of exposure and hypnozoite load might affect estimates of primaquine efficacy. Indeed, the relapse-free efficacy for heavily exposed battalion 2 receiving primaquine 15 mg in this study was only 27%, compared with 4% for dihydroartemisinin–piperaquine alone.

The adverse event profile for tafenoquine plus dihydroartemisinin–piperaquine was consistent with the known adverse event profiles for the individual drugs and there were no new safety signals. There were no deaths, and no patients withdrew from the study. Two patients had a serious adverse event, which the investigator considered related to study treatment, but neither received tafenoquine. QTc prolongation is a known risk with dihydroartemisinin–piperaquine.^31^ Co-administration of tafenoquine with dihydroartemisinin–piperaquine led to a small, asymptomatic increase in the incidence of QTc prolongation compared with dihydroartemisinin–piperaquine alone.^31^ This additional QTc prolongation effect of tafenoquine is not clinically relevant and has been observed previously in healthy volunteers.^17^ No patients had clinically significant reductions in haemoglobin and there were no reports of haemolysis. Increases in methaemoglobin concentrations were higher following primaquine plus dihydroartemisinin–piperaquine than tafenoquine plus dihydroartemisinin–piperaquine, but all were asymptomatic. An association between methaemoglobin and efficacy following treatment with primaquine^32^ has been previously noted; however, in post-hoc analysis combining data from this study and previous tafenoquine phase 3 studies, there was no significant association between maximum methaemoglobin and efficacy (p=0·257), for more information see the appendix 2 (p 26).

The current study has several limitations. It was only done in patients who had been infected with *P vivax* in Indonesian Papua, a region with one of the highest malaria transmission rates in the world, possibly associated with higher frequencies of hypnozoite carriage.^1^ The patient population was restricted to male Indonesian soldiers within a narrow age band. No pharmacokinetic data are yet available. However, other studies have found that tafenoquine pharmacokinetics are not influenced by age, sex, or ethnicity.^22^

The study design in which patients were infected in a highly malarious region and returned to an area free of malaria transmission meant that *P vivax* recurrences in the study were very probably due to relapse. However, it was not possible to know whether the absence of recurrent parasitaemia was due to efficacious treatment or simply natural hypnozoite depletion. Genetic homology is not validated as proof of whether recrudescence, reinfection, or relapse has occurred (a relapse can be either homologous or heterologous). Thus, as there are no definitive methods to establish the presence of hypnozoites in the liver or confirm whether *P vivax* parasitaemia is due to relapse or de novo re-infection, the model used in this study provides an optimal method of evaluating radical cure in the clinical setting.

In conclusion, the results of this study do not support co-administration of single 300-mg dose tafenoquine with dihydroartemisinin–piperaquine for the radical cure of *P vivax* malaria. Although primaquine plus dihydroartemisinin-piperaquine was more efficacious than tafenoquine plus dihydroartemisinin–piperaquine, nearly half of all patients receiving primaquine 15 mg daily for 14 days plus dihydroartemisinin–piperaquine also relapsed. The findings of this study do not alter the established favourable benefit–risk profile of single 300-mg dose tafenoquine co-administered with chloroquine for radical cure of *P vivax* malaria.

**This online publication has been corrected. The corrected version first appeared at thelancet.com/infection on July 25, 2023**

## Data sharing

Within 6 months of this publication, anonymised individual patient data, the annotated case report form, protocol, reporting and analysis plan, dataset specifications, raw dataset, analysis-ready dataset, and clinical study report will be available for research proposals approved by an independent review committee. Proposals should be submitted to https://www.clinicalstudydatarequest.com/. A data access agreement will be required.

## Declaration of interests

IS reports grants or contracts from Menzies School of Health Research, Darwin and the National Health and Medical Research Council**,** and funding from Medicines for Malaria Venture (MMV) and GSK. AS, KC, RN, DS, YWS, II, WB, CBP, and SL report that they or their institutions received funding from MMV to do the study and editorial support from GSK for this publication. LLE and AWS report institutional funding from Menzies School of Health Research, MMV, and GSK. CC-D reports institutional funding from MMV, GSK, and the Chan Zuckerberg Initiative. IE reports institutional funding from MMV, GSK, WHO, SPARK (University of Melbourne), and honoraria from the Indonesian Ministry of Health. SD is an employee of MMV; his institution received funding from ExxonMobil, the Bill & Melinda Gates Foundation (grant number INV-007155/19-BMGF-006), Newcrest Mining, and the UK Government to fund the INSPECTOR study. EC is a former independent contractor at GSK and holds shares in the company. EC developed the first draft of the manuscript and provided editorial services as an independent medical writer funded by GSK. KR, DF, AB, SJ, KF, HS, AM, MT, and LKT are employees of GSK and hold shares in the company. J-PK, NG, and JAG are former employees of GSK and hold shares in GSK. JKB reports institutional research funding from MMV, GSK, the Wellcome Trust, the Gates Foundation, FIND, Sanaria, UKAid, University of Oxford, US Centers for Disease Control and Prevention, and the Chinese Center for Disease Control; travel and speaking fees from the Belgian Society of Tropical Medicine, the International Conference of Tropical Medicine and Malariology, and Singapore Malaria Group Conference; and participation on the US National Health Institute Data Safety Monitoring Board.

## Supplementary Materials

Indonesian translation of the abstract

## References

[1] Battle KE, Karhunen MS, Bhatt S (2014). Geographical variation in *Plasmodium vivax* relapse. Malar J.

[2] Commons RJ, Simpson JA, Watson J, White NJ, Price RN (2020). Estimating the proportion of *Plasmodium vivax* recurrences caused by relapse: a systematic review and meta-analysis. Am J Trop Med Hyg.

[3] WHO (Nov 25, 2022). WHO Guidelines for malaria. https://www.who.int/teams/global-malaria-programme/guidelines-for-malaria.

[4] Douglas NM, Poespoprodjo JR, Patriani D (2017). Unsupervised primaquine for the treatment of *Plasmodium vivax* malaria relapses in southern Papua: a hospital-based cohort study. PLoS Med.

[5] Lacerda MVG, Llanos-Cuentas A, Krudsood S (2019). Single-dose tafenoquine to prevent relapse of *Plasmodium vivax* malaria. N Engl J Med.

[6] Llanos-Cuentas A, Lacerda MVG, Hien TT (2019). Tafenoquine versus primaquine to prevent relapse of *Plasmodium vivax* malaria. N Engl J Med.

[7] Price RN, von Seidlein L, Valecha N, Nosten F, Baird JK, White NJ (2014). Global extent of chloroquine-resistant *Plasmodium vivax*: a systematic review and meta-analysis. Lancet Infect Dis.

[8] Malaria Atlas Project (2022). Malaria atlas report. https://malariaatlas.org/trends/country/IDN.

[9] Sumawinata IW, Bernadeta, Leksana B (2003). Very high risk of therapeutic failure with chloroquine for uncomplicated *Plasmodium falciparum* and *P vivax* malaria in Indonesian Papua. Am J Trop Med Hyg.

[10] Sutanto I, Tjahjono B, Basri H (2013). Randomized, open-label trial of primaquine against vivax malaria relapse in Indonesia. Antimicrob Agents Chemother.

[11] Kementarian KRI (2020). Baku saku tatalaksana kasus malaria. https://drive.google.com/file/d/1lIkoYkKdl046AECgD-e6Jk3BNOIZu5Te/view.

[12] Gogtay N, Kannan S, Thatte UM, Olliaro PL, Sinclair D (2013). Artemisinin-based combination therapy for treating uncomplicated *Plasmodium vivax* malaria. Cochrane Database Syst Rev.

[13] Pasaribu AP, Chokejindachai W, Sirivichayakul C (2013). A randomized comparison of dihydroartemisinin-piperaquine and artesunate-amodiaquine combined with primaquine for radical treatment of vivax malaria in Sumatera, Indonesia. J Infect Dis.

[14] Elyazar IRF, Gething PW, Patil AP (2012). *Plasmodium vivax* malaria endemicity in Indonesia in 2010. PLoS One.

[15] Llanos-Cuentas A, Lacerda MV, Rueangweerayut R (2014). Tafenoquine plus chloroquine for the treatment and relapse prevention of *Plasmodium vivax* malaria (DETECTIVE): a multicentre, double-blind, randomised, phase 2b dose-selection study. Lancet.

[16] Ministry of Health Indonesia. 2018. Ministry of Health circular number HK.02.01/Menkes/584/2018 on acceleration of malaria reduction in malaria-endemic areas.

[17] Green JA, Mohamed K, Goyal N (2016). Pharmacokinetic interactions between tafenoquine and dihydroartemisinin-piperaquine or artemether-lumefantrine in healthy adult subjects. Antimicrob Agents Chemother.

[18] Baird JK, Louisa M, Noviyanti R (2018). Association of impaired cytochrome P450 2D6 activity genotype and phenotype with therapeutic efficacy of primaquine treatment for latent *Plasmodium vivax* malaria. JAMA Netw Open.

[19] Elyazar IR, Sinka ME, Gething PW (2013). The distribution and bionomics of anopheles malaria vector mosquitoes in Indonesia. Adv Parasitol.

[20] Watson JA, Commons RJ, Tarning J (2022). The clinical pharmacology of tafenoquine in the radical cure of *Plasmodium vivax* malaria: an individual patient data meta-analysis. eLife.

[21] Watson JA, Nekkab N, White M (2021). Tafenoquine for the prevention of *Plasmodium vivax* malaria relapse. Lancet Microbe.

[22] Thakkar N, Green JA, Koh GCKW, Duparc S, Tenero D, Goyal N (2018). Population pharmacokinetics of tafenoquine, a novel antimalarial. Antimicrob Agents Chemother.

[23] Vélez ID, Hien TT, Green JA (2022). Tafenoquine exposure assessment, safety, and relapse prevention efficacy in children with *Plasmodium vivax* malaria: open-label, single-arm, non-comparative, multicentre, pharmacokinetic bridging, phase 2 trial. Lancet Child Adolesc Health.

[24] Alving AS, Arnold J, Hockwald RS (1955). Potentiation of the curative action of primaquine in vivax malaria by quinine and chloroquine. J Lab Clin Med.

[25] Dembélé L, Franetich JF, Soulard V (2020). chloroquine potentiates primaquine activity against active and latent hepatic plasmodia *ex vivo*: potentials and pitfalls. Antimicrob Agents Chemother.

[26] Edgcomb JH, Arnold J, Yount EH (1950). Primaquine, SN 13272, a new curative agent in vivax malaria; a preliminary report. J Natl Malar Soc.

[27] Dow GS, Gettayacamin M, Hansukjariya P (2011). Radical curative efficacy of tafenoquine combination regimens in *Plasmodium cynomolgi*-infected Rhesus monkeys (Macaca mulatta). Malar J.

[28] Obaldia N, Rossan RN, Cooper RD (1997). WR 238605, chloroquine, and their combinations as blood schizonticides against a chloroquine-resistant strain of *Plasmodium vivax* in Aotus monkeys. Am J Trop Med Hyg.

[29] Collins WE, Jeffery GM (1996). Primaquine resistance in *Plasmodium vivax*. Am J Trop Med Hyg.

[30] Nelwan EJ, Ekawati LL, Tjahjono B (2015). Randomized trial of primaquine hypnozoitocidal efficacy when administered with artemisinin-combined blood schizontocides for radical cure of *Plasmodium vivax* in Indonesia. BMC Med.

[31] Funck-Brentano C, Bacchieri A, Valentini G (2019). Effects of dihydroartemisinin-piperaquine phosphate and artemether-lumefantrine on QTc interval prolongation. Sci Rep.

[32] Chu CS, Watson JA, Phyo AP (2021). Determinants of primaquine and carboxyprimaquine exposures in children and adults with *Plasmodium vivax* malaria. Antimicrob Agents Chemother.

