# Correction to *Lancet Infect Dis* 2023; published online May 23. https://doi.org/10.1016/S1473-3099(23)00213-X

**DOI:** 10.1016/S1473-3099(23)00489-9

**Published:** 2023-09

*Sutanto I, Soebandrio A, Ekawati LL, et al. Tafenoquine co-administered with dihydroartemisinin–piperaquine for the radical cure of* Plasmodium vivax *malaria (INSPECTOR): a randomised, placebo-controlled, efficacy and safety study.* Lancet Infect Dis *2023; published online May 23. https://doi.org/10.1016/S1473-3099(23)00213-X*—In this Article, the funding line should read “ExxonMobil, Bill & Melinda Gates Foundation, Newcrest Mining, UK Government all through Medicines for Malaria Venture; and GSK”; the copyright line should read “Copyright © The Author(s). Published by Elsevier Ltd. This is an Open Access article under the CC BY 4.0 license”; and the Acknowledgment and Declaration of interests regarding the funding received by MMV should read “…ExxonMobil and the Bill & Melinda Gates Foundation (grant number INV-007155/19-BMGF-006), Newcrest Mining, and the UK Government”. These corrections have been made to the online version as of July 25, 2023, and will be made to the printed version.

