# Supplementary material for: Tafenoquine co-administered with dihydroartemisinin–piperaquine for the radical cure of Plasmodium vivax malaria (INSPECTOR): a randomised, placebo-controlled, efficacy and safety study
Source: Lancet Infect Dis. 2023 Oct;23(10):1153–63. doi: 10.1016/S1473-3099(23)00213-X (PMC10533414; doi:10.1016/S1473-3099(23)00213-X)
Supplement: Indonesian translation of the abstract [file mmc1.pdf]

THE LANCET
Infectious Diseases

Supplementary appendix 1

This translation in Bahasa was submitted by the authors and we reproduce it as supplied. It has not been peer reviewed. *The Lancet*'s editorial processes have only been applied to the original in English, which should serve as reference for this manuscript.

Terjemahan dalam Bahasa Indonesia ini diserahkan oleh penulis dan diproduksi kembali apa adanya tanpa melalui proses peer review. Proses editorial Lancet hanya diterapkan pada versi original dalam Bahasa Inggris, yang harus dijadikan rujukan untuk naskah ini.

Supplement to: Authors. Tafenoquine co-administered with dihydroartemisinin–piperaquine for the radical cure of *Plasmodium vivax* malaria (INSPECTOR): a randomised, placebo-controlled, efficacy and safety study. *Lancet Infect Dis* 2023; published online May 23. https://doi.org/10.1016/S1473-3099(23)00213-X.

Ringkasan

Latar Belakang Tafenokuin, yang diberikan bersama klorokuin, disetujui untuk penyembuhan radikal (pencegahan kambuh) malaria *Plasmodium vivax*. Di daerah dengan resistensi klorokuin, terapi kombinasi berbasis artemisinin digunakan untuk mengobati malaria. Penelitian ini bertujuan mengevaluasi tafenokuin dengan terapi kombinasi berbasis artemisinin dihidroartemisinin-piperakuin untuk penyembuhan radikal malaria *P vivax*.

Metode Dalam penelitian uji klinis kelompok paralel, tersamar ganda, “double-dummy”, tentara Indonesia dengan glukosa-6-fosfat normal dan terinfeksi malaria *P vivax* yang dikonfirmasi secara mikroskopis dipillih secara acak menggunakan tabel randomisasi dari komputer (1:1:1) ke dalam kelompok dihidroartemisinin-piperakuin saja, dihidroartemisinin-piperakuin ditambah tafenokuin dosis tunggal 300 mg, atau dihidroartemisinin-piperakuin ditambah primakuin (15mg) selama 14 hari. Luaran utama adalah efikasi tafenokuin dikombinasi dengan dihidroartemisinin-piperakuin dibandingkan dengan dihidroartemisinin-piperakuin saja dalam mencegah kekambuhan selama 6 bulan pada semua pasien yang secara acak menerima setidaknya satu dosis pengobatan plasebo dan dikonfirmasi secara mikroskopis memiliki *P. vivax* pada awal penelitian (*microbiological intention to treat population*). Keamanan pasien adalah luaran sekunder dan keamanan populasi terdiri dari semua pasien yang menerima setidaknya satu dosis obat plasebo. Penelitian ini terdaftar di ClinicalTrials.gov, NTC02802501 dan telah selesai dilaksanakan.

Hasil Antara 8 April 2018 dan 4 Februari 2019, dari 164 pasien yang diskrining untuk kelayakan, 150 dipilih secara acak (50 per kelompok terapi). Analisis Kaplan-Meier menunjukkan bahwa efikasi bebas kambuh dalam waktu 6 bulan (*microbial intention to treat*) adalah 11% (95% CI 4-22) pada pasien yang diobati dengan dihidroartemisinin-piperakuin saja versus 21% (11-34) pada pasien yang diobati dengan tafenokuin plus dihidroartemisinin-piperakuin (hazard ratio 0,44; 95% CI [0,29-0,69]) dan 52% (37-65) pada kelompok primakuin plus dihidroartemisinin-piperakuin. Kejadian yang tidak diinginkan selama 28 hari pert.ama dilaporkan pada 27 (54%) dari 50 pasien yang diobati dengan dihidroartemisinin-piperakuin saja, 29 (58%) pasien yang diobati dengan tafenokuin plus dihidroartemisinin-piperakuin, dan 22 (44%) dari 50 pasien yang diobati dengan primakuin plus dihidroartemisinin-piperakuin. Kejadian yang tidak diinginkan serius dilaporkan pada satu (2%) dari 50, dua (4%) dari 50, dan dua (4%) dari 50 pasien, di masing-masing kelompok terapi.

Interpretasi Meskipun tafenokuin plus dihidroartemisinin-piperakuin secara statistik lebih unggul dalam penyembuhan radikal malaria *P vivax* daripada dihidroartemisinin-piperakuin saja, manfaatnya tidak bermakna secara klinis. Ini berbeda dengan penelitian sebelumnya, di mana tafenokuin plus klorokuin secara klinis lebih unggul dibandingkan dengan klorokuin saja dalam menyembuhkan malaria *P vivax* secara radikal.
