# Supplementary material for: Tafenoquine co-administered with dihydroartemisinin–piperaquine for the radical cure of Plasmodium vivax malaria (INSPECTOR): a randomised, placebo-controlled, efficacy and safety study
Source: Lancet Infect Dis. 2023 Oct;23(10):1153–63. doi: 10.1016/S1473-3099(23)00213-X (PMC10533414; doi:10.1016/S1473-3099(23)00213-X)
Supplement: Supplementary appendix 2 [file mmc2.pdf]

THE LANCET
Infectious Diseases

Supplementary appendix 2

This appendix formed part of the original submission and has been peer reviewed. We post it as supplied by the authors.

Supplement to: Authors. Tafenoquine co-administered with dihydroartemisinin–piperaquine for the radical cure of *Plasmodium vivax* malaria (INSPECTOR): a randomised, placebo-controlled, efficacy and safety study. *Lancet Infect Dis* 2023; published online May 23. https://doi.org/10.1016/S1473-3099(23)00213-X.

Supplementary Appendix

Randomised, Placebo-Controlled, Efficacy and Safety Study of Tafenoquine Co-administered With Dihydroartemisinin-piperaquine for the Radical Cure of *Plasmodium vivax* Malaria (INSPECTOR)

Contents

Study inclusion and exclusion criteria 3

Additional Methods 6

Table S1. Demographic and baseline characteristics by battalion 7

Table S2. Survival Analysis of Relapse-Free Efficacy at 6 Months – Per-Protocol Population 9

Table S3. Summary of Covariate and Treatment by Covariate Interaction for Cox Proportional Hazards: Model of Relapse-Free Efficacy over 6 Months 10

Table S4. Summary of Relapse-Free Efficacy at 6 Months by Weight Category (mITT Population; Post-Hoc Analysis) 11

Table S5. Logistic Regression Analysis of Relapse-Free Efficacy at 6 Months by Battalion – Tafenoquine versus Primaquine Comparison (mITT Population; Post-Hoc Analysis) 12

Table S6. Survival Analysis of Relapse-Free Efficacy over Four Months (mITT Population) 13

Table S7. Time to Parasite Clearance and Fever Clearance (mITT Population) 14

Table S8. Logistic Regression Analysis of Relapse-Free Efficacy at 6 Months by CYP2D6 Activity Score (mITT Population, Post-Hoc Analysis) 15

Table S9. Logistic Regression Analysis of Relapse-Free Efficacy at 6 months by CYP2D6 Metaboliser Class (mITT Population, Post-Hoc Analysis) 16

Table S10. Summary of All Adverse Events During the Double-Blind Treatment Phase, by Preferred Term and Frequency (Safety Population) 17

Table S11. Adverse Events of Special Interest During the Double-Blind Treatment Phase (Safety Population) 19

Table S12. Post-baseline Change in QTcF Values (Safety Population) 20

Figure S1. Map of Endemicity of *Plasmodium Vivax* in Indonesia in 2010 (Elyazar, 2012) 21

Figure S2. Map of Java Showing the Study Site Locations at the Two Army Bases GEOSS PROTAL European Space Agency 21

Figure S3. Study 200894 Design Schematic 22

Figure S4. Kaplan-Meier Survival Curves for Time to Relapse by Battalion (mITT Population) 23

Figure S5. Incidence (95% CI) of Genetically Heterologous and Homologous Relapses 24

Figure S6. Common (≥5% in any Treatment Group) AEs and Relative Risk During the Double-Blind Treatment Phase (Safety Population) 25

Figure S7. Estimated Probability of Being Relapse-Free at 4 Months by Maximum Methaemoglobin A) by Treatment Group and B) Combined From the DETECTIVE Part 1, DETECTIVE Part 2, GATHER and INSPECTOR Studies 26

Supplementary References 28

Study inclusion and exclusion criteria

Inclusion Criteria

A patient will be eligible for inclusion in this study only if all of the following criteria apply:

AGE and SEX
1. Male patients ≥18 years at the time of signing the informed consent.
TYPE OF PATIENT AND DIAGNOSIS INCLUDING DISEASE SEVERITY
2. The patient has a positive Giemsa smear for *P. vivax* (mixed infection with *P. falciparum* is acceptable).<br><br>3. The patient has a parasite density of >20/µL.<br><br>4. Glucose-6-phosphate dehydrogenase (G6PD) normal using a suitable qualitative assessment; eg, NADPH qualitative fluorescent spot test (Trinity Biologicals, USA).<br><br>5. The patient has a QTcF of <450 msec.<br><br>*N.B.* Reading based on an average of triplicate ECGs obtained over a brief recording period by machine.<br><br>6. The patient is willing and able to comply with the study protocol.
INFORMED CONSENT
7. Capable of giving signed informed consent which includes compliance with the requirements and restrictions listed in the consent form and in this protocol.

Exclusion Criteria

A patient will not be eligible for inclusion in this study if any of the following criteria apply:

CONCURRENT CONDITIONS/MEDICAL HISTORY (INCLUDES LIVER FUNCTION AND QTc INTERVAL)
1. Severe *P. vivax* malaria as defined by WHO criteria.<br><br>2. Severe vomiting (no food or inability to take food during the previous 8 hours).<br><br>3. Screening haemoglobin (Hb) concentration <8g/dL.<br><br>4. Liver function test ALT >2xULN.<br><br>5. Any clinically significant concurrent illness (eg, pneumonia, septicaemia), significant pre-existing conditions (eg, renal disease, malignancy, type 1 diabetes), conditions that may affect absorption of study treatment (eg, vomiting, severe diarrhoea), or clinical signs and symptoms of severe cardiovascular disease (eg, uncontrolled congestive heart failure, severe coronary artery disease). These abnormalities may be identified on the screening history and physical or laboratory examination.<br><br>6. History of hypersensitivity, allergy or adverse reactions to dihydroartemisinin (DHA) or other artemisinins, piperaquine, tafenoquine or primaquine.
CONCOMITANT MEDICATIONS

7. Patient has previously received treatment with tafenoquine or has received treatment with any other investigational drug within 30 days of study entry or within 5 half-lives, whichever is longer.

8. Patient has taken antimalarials (eg, ACTs, mefloquine, primaquine, quinacrine) or drugs with antimalarial activity within the past 30 days.

9. Patients who will likely require the use of medications from the prohibited medications list or have taken them in the past 30 days which include the following medications and medication classes:

    - Drugs with haemolytic potential

    - Drugs known to prolong the QTc interval including:

        - Antiarrhythmics (eg, amiodarone, disopyramide, dofetilide, ibutilide, procainamide, quinidine, hydroquinidine, sotalol).

        - Neuroleptics (eg, phenothiazines, sertindole, sultopride, chlorpromazine, haloperidol, mesoridazine, pimozide, or thioridazine) and antidepressive agents.

        - Certain antimicrobial agents, including agents of the following classes: macrolides (eg, erythromycin, clarithromycin), fluroquinolones (eg, moxifloxacin, sparfloxacin), imidazole and triazole antifungal agents and also pentamidine and saquinavir.

        - Certain non-sedating antihistamines (eg, terfenadine, astemizole, mizolastine).

        - Cisapride, droperidol, domperidone, bepridil, diphemanil, probucol, levomethadyl, methadone, vinca alkaloids, arsenic trioxide.

    - The biguanides: phenformin and buformin (but excluding metformin).

    - Drugs that are substrates of the renal transporters OCT2, MATE1 and MATE2 and have a narrow therapeutic index (for example, the antiarrhythmic agents: dofetilide, procainamide and pilsicainide).

RELEVANT HABITS

10. Anticipated to be unable to consume daily study treatment under direct supervision by the research team.

11. Previous participation in the present clinical trial; ie, patients experiencing relapse during or after the study period may not be enrolled as a new patient.

12. History of illicit drug abuse or heavy alcohol intake, such that full participation in the study could be compromised.

CONTRAINDICATIONS

13. Any contraindication in the opinion of the Investigator to DP or primaquine administration (refer to locally approved prescribing information for primaquine) such as:

    - Family history of sudden unexplained death (DP)

    - Known congenital QTc prolongation (DP)

    - Known history of a medical condition known to prolong the QT interval; eg, myxoedema, cardiomyopathies, recent myocardial infarction (DP)

    - History of symptomatic cardiac arrhythmias or with clinically relevant bradycardia (DP)

- Cardiac illnesses predisposing to arrhythmias; eg, severe hypertension, left ventricular hypertrophy, cardiomyopathies, cardiac failure with reduced ejection fraction (DP)
- Presence of an electrolyte disturbance particularly hypokalaemia, hypocalcaemia, hypomagnesemia (DP)
- Rheumatoid arthritis, lupus erythematosus and other systemic conditions that may cause granulocytopenia (primaquine)
- History of haemolytic anaemia, methemoglobinemia and leucopoenia (primaquine)

DP; dihydroartemisinin-piperaquine.

Additional Methods

Slide Preparation

Four slides (two thick films and one thin film, plus an additional unstained slide with both thick and thin films for contingency) were prepared at each time point. The two thick film slides were used to determine the parasite counts. The thin film slide was only examined in case of heavy infection (>250 parasites per 50 WBC, and/or to confirm speciation). Slides were stained with 3% Giemsa for 45–60 minutes.

Blood films were prepared pre-dose and once post-dose on day 1, and then twice daily on days 2 and 3, 6–12 hours apart. Where a patient received dihydroartemisinin-piperaquine <6 hours from midnight on day 1, the post-dose day 1 slide could be omitted. Additionally, blood films were obtained whenever parasitological reassessment was required and at any relapse visit or withdrawal visit.

Malaria Identification, Quantification and Quality Control

All staff reading microscopy slides were required to undergo training provided by Family Health International (FHI 360) to certify proficiency prior to participation in the study. Two certified site microscopists independently determined the parasite counts and the mean values were recorded. Any discrepancies (greater than a pre-specified limit) were resolved by a third certified site microscopist counting the slides. The parasite count of the first two microscopists that was closest to that of the third microscopist was documented. The pre-dose smear was used to calculate the parasite density of enrolled patients. Slides were considered negative after review of 100 high-power fields. All baseline, relapse, and a sample of other post-baseline slides were examined by a blinded external laboratory (FHI 360, Bangkok, Thailand) for quality assurance.

Parasite Genetics

Parasite DNA extraction and PCR analysis was performed on a blood sample collected at screening and at the first relapse to determine the proportion of heterologous and homologous relapses. Five microsatellite markers (*Pv*1F3, *Pv*MS16, *Pv*MS2, *Pv*MS3.27 and *Pv*MS20) were used to distinguish between genetically homologous and heterologous relapses. Blinded external quality assurance was performed on 20 paired samples for parasite DNA analysis at Walter and Eliza Hall Institute, Melbourne, Australia.

CYP2D6 Analysis

Blood samples were collected at screening for cytochrome P-450 2D6 (CYP2D6) genotyping and analysed using xTAG® CYP2D6 kit version 3 (Luminex® Corporation, USA) as per manufacturer's instructions. The CYP2D6 activity score system assigns an activity value to both alleles. The sum of these values is used to predict CYP2D6 metabolic activity. The CYP2D6 phenotype was prospectively determined using the Dutch Pharmacogenomics Working Group classification scheme (poor metaboliser: score 0, intermediate metaboliser: score 0.5 or 1, extensive metaboliser: 1.5 or 2, ultra-metaboliser: score ≥2.5) [Gaedigk, 2008]. This was subsequently revised as a post-hoc analysis using the updated CPIC guidelines to assign the CYP2D6 activity score and phenotype classification (poor metaboliser: score 0, intermediate metaboliser: score 0.25 to 1.0, normal metaboliser: score 1.25 to 2.25, ultra-metaboliser: score >2.25) [Caudle, 2020]. Blinded external quality assurance was performed on 20% of samples for CYP2D6 analysis at Indiana University, USA.

Pharmacokinetic Analysis

Blood samples for tafenoquine pharmacokinetic analysis were taken pre-dose, 6–12 hours and 24–48 hours post-blinded study treatment and then on day 7, 14, 28, 60 or at relapse (up to 60 days post-dose). Samples were then stored at −80°C until analysis could be undertaken.

Additional Post-Hoc Analyses

Additional post-hoc analyses were performed to assess, separately, the impact of weight and methaemoglobin on efficacy (relapse). The effect of weight was assessed by summarising relapse-free efficacy at 6 months for participants from the INSPECTOR study with weights above and below the median baseline weight (69 kg). The effect of a participant's maximum methaemoglobin was assessed using combined data from the INSPECTOR, DETECTIVE parts 1 and 2, and GATHER studies. A logistic regression model was fitted for relapse at 4 months, including covariates for study, treatment and individual participant's maximum methaemoglobin. In addition to assessing the statistical significance of the maximum methaemoglobin covariate, the model was used to estimate the probability of being relapse-free at 4 months for different maximum methaemoglobin levels, for each treatment group and overall. These probabilities, including the 95% confidence interval for the overall probability of relapse, were then plotted.

Table S1. Demographic and Baseline Characteristics by Battalion

Battalion 1

	DP alone (N=23)	Tafenoquine+DP (N=22)	Primaquine+DP (N=24)
**Age (years)**			
Mean (SD)	28·3 (4·02)	30·6 (5·97)	28·3 (4·75)
Median (Min, Max)	29·0 (21, 35)	29·5 (21, 41)	28·0 (22, 38)
**Sex, n (%)**			
Male	23 (100)	22 (100)	24 (100)
**Geographic ancestry, n (%)**			
Southeast Asian Heritage	23 (100)	22 (100)	24 (100)
**Weight (kg)**			
Mean (SD)	69·6 (9·56)	67·2 (8·89)	70·7 (9·17)
Median (Min, Max)	67·0 (54·0, 88·5)	67·3 (55·0, 86·5)	69·5 (59·0, 95·5)
**Height (cm)**			
Mean (SD)	167·7 (4·04)	168·8 (3·32)	168·9 (4·14)
Median (Min, Max)	166·0 (163, 176)	168·0 (163, 174)	168·0 (163, 176)
**Body mass index (kg/m^2)**			
Mean (SD)	24·8 (3·65)	23·6 (3·10)	24·8 (2·79)
Median (Min, Max)	24·4 (18·9, 33·3)	23·0 (19·8, 31·4)	24·5 (20·5, 31·9)
**G6PD enzyme activity (IU/g Hb)**			
Mean (SD)	7·68 (1·42)	7·80 (1·04)	7·81 (1·34)
Median (Min, Max)	7·22 (5·77, 12·75)	7·68 (5·80, 10·35)	7·55 (6·13, 11·92)

Battalion 2

	DP alone (N=27)	Tafenoquine+DP (N=28)	Primaquine+DP (N=26)
**Age (years)**			
Mean (SD)	28·3 (4·68)	28·4 (4·18)	28·8 (6·30)
Median (Min, Max)	28·0 (21, 42)	28·0 (22, 34)	28·0 (22, 49)
**Sex, n (%)**			
Male	27 (100)	28 (100)	26 (100)
**Geographic ancestry, n (%)**			
Southeast Asian Heritage	27 (100)	28 (100)	26 (100)
**Weight (kg)**			
Mean (SD)	72·0 (10·24)	71·6 (12·10)	70·1 (8·73)
Median (Min, Max)	71·0 (54·0, 97·0)	71·0 (57·0, 114·0)	70·0 (55·0, 94·0)
**Height (cm)**			
Mean (SD)	171·0 (3·39)	171·3 (3·22)	169·7 (3·39)
Median (Min, Max)	171·0 (164, 179)	171·0 (165, 178)	169·5 (165, 178)
**Body mass index (kg/m^2)**			
Mean (SD)	24·7 (3·63)	24·4 (4·28)	24·3 (2·51)
Median (Min, Max)	24·2 (18·7, 35·2)	23·5 (19·5, 39·5)	23·9 (19·7, 29·7)
**G6PD enzyme activity (IU/g Hb)**			
Mean (SD)	7·85 (1·02)	7·60 (0·92)	7·50 (0·84)
Median (min, max)	7·62 (6·14, 10·10)	7·44 (6·53, 10·74)	7·58 (6·42, 10·19)

G6PD, glucose-6-phosphate dehydrogenase; SD, standard deviation.

Table S2. Survival Analysis of Relapse-Free Efficacy at 6 Months – Per-Protocol Population

	DP alone (N=44)	Tafenoquine+DP (N=40)	Primaquine+DP (N=42)
Patients observed to relapse prior to or at 6 months, n (%)	39 (89)	35 (88)	23 (55)
Censored, relapse free at 6 months, n (%)	5 (11)	5 (13)	19 (45)
**Relapse-free efficacy rate at 6 months**			
Estimate (95% CI)	10·2% (3·2, 22·0)	11·3% (3·5, 24·0)	45·2% (29·9, 59·4)
**Hazard ratio of risk of relapse vs DP alone**			
Estimate (95% CI)		0·501 (0·312, 0·804)	0·301 (0·177, 0·512)

CI, confidence interval; DP, dihydroartemisinin-piperaquine.

Table S3. Summary of Covariate and Treatment by Covariate Interaction for Cox Proportional Hazards: Model of Relapse-Free Efficacy over 6 Months

Terms in the model	Freedom	Chi-Square	*P* value
Battalion	1	32·96	<0·0001
Treatment*battalion	2	5·64	0·060
Baseline asexual Parasite Count	1	0·02	0·887
Treatment*baseline asexual parasite count	2	1·28	0·527
Baseline weight	1	0·05	0·831
Treatment*baseline weight	2	1·31	0·520

Battalion: Model fitted first with battalion and treatment, then with battalion, treatment and treatment by battalion interaction.

Parasite count and weight: Model fitted first with covariate, battalion and treatment, then with covariate, battalion, treatment and treatment by covariate interaction.

Table S4. Summary of Relapse-Free Efficacy at 6 Months by Weight Category (mITT Population; Post-Hoc Analysis)

	DP alone	Tafenoquine+DP	Primaquine+DP
**Patients weight ≤69 kg**	**N=23**	**N=27**	**N=25**
Patients with relapse-free efficacy at 6 months (primary analysis)	3 (13%)	9 (33%)	16 (64%)
Patients that did not demonstrate initial clearance of *P. vivax* parasitaemia	0	0	0
Patients with recurrence of parasitaemia in 6 months after initial clearance	20 (87%)	18 (67%)	9 (36%)
Patients who took drug with antimalarial action in the first 6 months and were not parasitaemic	0	0	0
Patients who were not confirmed parasite-free at 6-month assessment	0	0	0
**Patients weight >69 kg**	**N=27**	**N=23**	**N=25**
Patients with relapse-free efficacy at 6 months (primary analysis)	3 (11%)	2 (9%)	10 (40%)
Patients that did not demonstrate initial clearance of *P. vivax* parasitaemia	0	0	0
Patients with recurrence of parasitaemia in 6 months after initial clearance	24 (89%)	21 (91%)	15 (60%)
Patients who took drug with antimalarial action in the first 6 months and were not parasitaemic	0	0	0
Patients who were not confirmed parasite-free at 6-month assessment	0	0	0

DP, dihydroartemisinin-piperaquine; mITT, modified intent to treat.

Table S5. Logistic Regression Analysis of Relapse-Free Efficacy at 6 Months by Battalion – Tafenoquine versus Primaquine Comparison (mITT Population; Post-Hoc Analysis)

				Comparison with primaquine+DP	
Treatment	N	Patients relapse free, n (%)	Patients relapsed n (%)	Adjusted Odds Ratio of Relapse[a]	95% CI
**Battalion 1**					
DP alone	23	5 (22)	18 (78)	---	---
Tafenoquine+DP	22	7 (32)	15 (68)	8·14	(2·15, 30·86)
Primaquine+DP	24	19 (79)	5 (21)	---	---
**Battalion 2**					
DP alone	27	1 (4)	26 (96)	---	---
Tafenoquine+DP	28	4 (14)	24 (86)	2·21	(0·56, 8·68)
Primaquine+DP	26	7 (27)	19 (73)	---	---

CI, confidence interval; DP, dihydroartemisinin-piperaquine; mITT, modified intent to treat.

[a] An odds ratio <1 represents a smaller chance of relapse compared to primaquine+DP.

Patients who did not demonstrate initial clearance of *P. vivax* parasitaemia, take a concomitant medication with antimalarial activity, have a missing day 180 assessment, or have a zero *P. vivax* asexual parasite count at baseline were to be excluded from the analysis. No patients met these criteria.

Table S6. Survival Analysis of Relapse-Free Efficacy over Four Months (mITT Population)

	DP alone (N=50)	Tafenoquine+DP (N=50)	Primaquine+DP (N=50)
**Number of patients, n (%)**			
Relapse-free at 4 months	8 (16)	14 (28)	29 (58)
Relapse prior to or at 4 months	42 (84)	36 (72)	20 (40)
Censored, prior to 4-month assessment[a]	0	0	1 (2)
**Relapse-free efficacy rate at 4 months[b]**			
Estimate (95% CI)	14·0 (5·7, 25·9)	10·8[c] (1·2, 32·7)	59·2 (44·2, 71·4)
**Hazard ratio of risk of relapse vs DP alone[d]**			
Estimate (95% CI)	---	0·433 (0·273, 0·686)	0·245 (0·142, 0·423)
P value	---	0.0004	<0·0001
**Hazard ratio of risk of relapse vs primaquine+DP[d]**			
Estimate (95% CI)	---	1·769 (1·020, 3·067)	NA

CI, confidence interval; DP, dihydroartemisinin-piperaquine; mITT, modified intent to treat.

[a] Patients were to be censored if did not demonstrate initial clearance of *P. vivax* parasitaemia, took a drug with antimalarial action despite not having malaria parasites, or did not have a 4-month assessment.
[b] Kaplan-Meier methodology.
[c] The Kaplan-Meier relapse-free efficacy estimate in the tafenoquine+DP group appears artificially low due to the nature of the censoring at this time point; therefore, this result should be interpreted with caution.
[d] Estimated from Cox's Proportional Hazards Analysis, adjusting for Battalion. A hazard ratio <1 indicates a lower chance of relapse compared to the reference treatment.

Table S7. Time to Parasite Clearance and Fever Clearance (mITT Population)

	DP alone (N=50)	Tafenoquine+DP (N=50)	Primaquine+DP (N=50)
**Asexual parasite clearance, n (%)**			
Asexual parasite clearance achieved	50 (100)	50 (100)	50 (100)
Censored, asexual parasite clearance not achieved	0 (0)	0 (0)	0 (0)
**Time to asexual parasite clearance (hours)[a]**			
Median (95% CI)	18·1 (18·0, 18·7)	18·1 (18·0, 18·3)	18·0 (18·0, 18·1)
**Gametocyte clearance, n (%)**			
Gametocyte at baseline	42 (84)	40 (80)	39 (78)
Gametocyte clearance achieved	42 (84)	40 (80)	39 (78)
Censored, gametocyte clearance not achieved[b]	8 (16)	10 (20)	11 (22)
**Time to gametocyte clearance (hours)[a]**			
Median (95% CI)	25·6 (18·1, 28·6)	23·9 (18·2, 25·7)	21·7 (18·1, 24·4)
**Fever clearance, n (%)**			
Fever at Baseline	28 (56)	20 (40)	27 (54)
Fever clearance achieved	28 (56)	20 (40)	27 (54)
Censored, fever clearance not achieved[c]	22 (44)	30 (60)	23 (46)
**Time to fever clearance (hours)[c]**			
Median (95% CI)	16·5 (14·1, 17·3)	15·8 (13·1, 18·8)	16·8 (14·6, 17·8)

CI, confidence interval; DP, dihydroartemisinin-piperaquine; mITT, modified intent to treat.

[a] Kaplan-Meier methodology.
[b] Patients were censored at baseline if they had a negative gametocyte count or at their last nonmissing assessment.
[c] Patients were censored if baseline temperature was not >37·4°C or if the method of assessment was not consistent. Patients not demonstrating this endpoint prior to study day 3 were censored at that time point.

Table S8. Logistic Regression Analysis of Relapse-Free Efficacy at 6 Months by CYP2D6 Activity Score (mITT Population, Post-Hoc Analysis)

CYP2D6 Activity Score[a]	DF	Chi-Square	*P* value
DP alone	1	0·43	0·512
Tafenoquine+DP	1	0·19	0·665
Primaquine+DP	1	0·65	0·421

DF, degrees of freedom; DP, dihydroartemisinin-piperaquine; mITT, modified intent to treat.

[a] Model fitted with CYP2D6 Activity Score separately for each treatment.
Note: *P* value from a 2-sided test presented. The effect of CYP2D6 activity score on relapse-free efficacy at 6 months by logistic regression was not significant in any treatment group.

Table S9. Logistic Regression Analysis of Relapse-Free Efficacy at 6 months by CYP2D6 Metaboliser Class (mITT Population, Post-Hoc Analysis)

Treatment group effect tested	N	Patients relapse free, n (%)	Adjusted odds ratio vs extensive metaboliser+ultra metaboliser	Lower limit of 90% CI	*P* value
**DP alone**					
Poor or intermediate metaboliser	24	2 (8)	0·500	0·083	0·225
Normal or ultra metaboliser	26	4 (15)			
**Tafenoquine+DP**					
Poor or intermediate metaboliser	23	6 (26)	1·553	0·405	0·739
Normal or ultra metaboliser	27	5 (19)			
**Primaquine+DP**					
Poor or intermediate metaboliser	20	10 (50)	0·875	0·282	0·409
Normal or ultra metaboliser	30	16 (53)			

CI, confidence interval; DP, dihydroartemisinin-piperaquine; mITT, modified intent to treat.

Model includes term for CYP2D6 metaboliser class. Metaboliser class is determined from the CYP2D6 activity score (AS) as follows: Poor AS=0; Intermediate AS=0·25 to 1; Normal AS=1·25 to 2·25; Ultra AS >2·25 (Caudle 2020). The effect of CYP2D6 metaboliser class on relapse-free efficacy at 6 months by logistic regression was not significant in any treatment group.

Table S10. Summary of All Adverse Events During the Double-Blind Treatment Phase, by Preferred Term and Frequency (Safety Population)

	DP alone		Tafenoquine+DP		Primaquine+DP	
**Preferred term**	**(N=50)**		**(N=50)**		**(N=50)**	
Any event	41	(82%)	41	(82%)	34	(68%)
Upper respiratory tract infection	7	(14%)	11	(22%)	8	(16%)
Electrocardiogram QT prolonged	4	(8%)	6	(12%)	2	(4%)
Myalgia	2	(4%)	6	(12%)	7	(14%)
Nasopharyngitis	7	(14%)	5	(10%)	2	(4%)
Pharyngitis	0		5	(10%)	2	(4%)
Vomiting	6	(12%)	5	(10%)	1	(2%)
Headache	3	(6%)	4	(8%)	8	(16%)
Nausea	5	(10%)	4	(8%)	3	(6%)
Rhinitis	3	(6%)	4	(8%)	2	(4%)
Chills	0		3	(6%)	0	
Dyspepsia	2	(4%)	3	(6%)	4	(8%)
Arthralgia	1	(2%)	2	(4%)	1	(2%)
Diarrhoea	5	(10%)	2	(4%)	2	(4%)
Dizziness	1	(2%)	2	(4%)	1	(2%)
Malaise	1	(2%)	2	(4%)	3	(6%)
Skin abrasion	0		2	(4%)	1	(2%)
Abdominal distension	1	(2%)	1	(2%)	1	(2%)
Asthenia	3	(6%)	1	(2%)	1	(2%)
Back pain	2	(4%)	1	(2%)	0	
Bradycardia	0		1	(2%)	0	
Chronic gastritis	0		1	(2%)	0	
Constipation	0		1	(2%)	0	
Cough	2	(4%)	1	(2%)	1	(2%)
Decreased appetite	2	(4%)	1	(2%)	0	
Dermatitis contact	0		1	(2%)	2	(4%)
Drug-induced liver injury	0		1	(2%)	0	
Eczema nummular	0		1	(2%)	0	
Flank pain	0		1	(2%)	0	
Folliculitis	0		1	(2%)	0	
Furuncle	0		1	(2%)	0	
Gastroenteritis	1	(2%)	1	(2%)	2	(4%)
Gingival pain	0		1	(2%)	0	
Hand fracture	0		1	(2%)	0	
Hepatic enzyme increased	1	(2%)	1	(2%)	0	
Hepatitis	0		1	(2%)	0	
Hypochromic anaemia	0		1	(2%)	0	
Pharyngotonsillitis	0		1	(2%)	0	
Pulpitis dental	0		1	(2%)	0	
Respiratory tract infection	0		1	(2%)	0	
Sciatica	0		1	(2%)	0	
Typhoid fever	0		1	(2%)	0	
Abdominal discomfort	0		0		2	(4%)
Alanine aminotransferase increased	1	(2%)	0		0	
Arthropod bite	1	(2%)	0		0	
Aspartate aminotransferase increased	1	(2%)	0			
Bacterial infection	0		0		1	(2%)
Cellulitis	1	(2%)	0		0	
Chest pain	0	0		1		(2%)
Cholelithiasis	0	0		1		(2%)
Conjunctival hyperaemia	0	0		1		(2%)
Conjunctivitis bacterial	1	(2%)	0	0		
Conjunctivitis viral	1	(2%)		0	0	
Dysentery	0	0		1		(2%)
Erysipelas	0	0		1		(2%)
Eye irritation	0	0		1		(2%)
Eyelid oedema	1	(2%)	0	0		
Fatigue	1	(2%)	0	0		
Gastritis	0		0	2		(4%)

Haemorrhoidal haemorrhage	0		0	1		(2%)
Hordeolum	1	(2%)	0	1		(2%)
Hyperkeratosis	1	(2%)	0			
Hypertensive urgency	0		0			
Influenza	1	(2%)	0		1	(2%)
Irritable bowel syndrome	2	(4%)	0		0	
Laryngitis	0	0	0		1	(2%)
Ligament sprain	0	0			1	(2%)
Normochromic normocytic anaemia	1	(2%)	0		0	
Palpitations	1	(2%)	0		0	
Periodontitis	1	(2%)	0		0	
Pyrexia	2	(4%)	0		1	(2%)
Sinus arrhythmia	0		0		1	(2%)
Skin bacterial infection	1	(2%)	0		0	
Skin laceration	0		0		2	(4%)
Stomatitis	0		0		1	(2%)
Supraventricular extrasystoles	0		0		1	(2%)
Tendonitis	0		0		1	(2%)
Tension headache	0		0		2	(4%)
Torticollis	1	(2%)	0		0	
Upper respiratory tract infection bacterial	0	0			1	(2%)
Ventricular extrasystoles	1	(2%)	0		0	
Viral infection	2	(4%)	0		0	
Wound	1	(2%)	0		1	(2%)

DP, dihydroartemisinin-piperaquine.

Table S11. Adverse Events of Special Interest During the Double-Blind Treatment Phase (Safety Population)

Preferred term	DP alone (N=50)		Tafenoquine+DP (N=50)		Primaquine+DP (N=50)	
**Any event**	6	(12%)	9	(18%)	10	(20%)
**Nervous system disorders**						
Any Event	4	(8%)	7	(14%)	9	(18%)
Headache	3	(6%)	4	(8%)	8	(16%)
Dizziness	1	(2%)	2	(4%)	1	(2%)
Sciatica	0		1	(2%)	0	
Tension headache	0		0		2	(4%)
**Hepatobiliary disorders**						
Any event	0		1	(2%)	1	(2%)
Drug-induced liver injury	0		1	(2%)	0	
Hepatitis	0		1	(2%)	0	
Cholelithiasis	0		0		1	(2%)
**Hematologic events of special interest**						
Any event	2	(4%)	1	(2%)	0	
Blood and lymphatic system disorders						
Any event	1	(2%)	1	(2%)	0	
Hypochromic aneaemia	0		1	(2%)	0	
Nomochromic nomocytic anaemia	1	(2%)	0		0	
General disorders and administration site conditions						
Any event	1	(2%)	0		0	
Fatigue	1	(2%)	0		0	

DP, dihydroartemisinin-piperaquine.

Table S12. Post-baseline Change in QTcF Values (Safety Population)

Assessment window<br>QTcF Post-baseline change (msec)	DP alone (N=50), n (%)	Tafenoquine +DP (N=50), n (%)	Primaquine +DP (N=50), n (%)
**Maximum change during the study**			
N	50	50	50
Increase 0 to <30	17 (34)	11 (22)	13 (26)
Increase ≥30 to <60	26 (52)	27 (54)	32 (64)
Increase ≥60 and QTcF≤480	5 (10)	9 (18)	4 (8)
Increase ≥60 and QTcF>480	2 (4)	3 (6)	1 (2)
**Day 3: 4 hour post-DP dose**			
N	50	50	50
Increase 0 to <30	16 (32)	11 (22)	15 (30)
Increase ≥30 to <60	25 (50)	26 (52)	30 (60)
Increase ≥60 and QTcF≤480	5 (10)	9 (18)	4 (8)
Increase ≥60 and QTcF>480	2 (4)	3 (6)	1 (2)
**Day 7**			
N	50	50	50
Increase 0 to <30	35 (70)	35 (70)	34 (68)
Increase ≥30 to <60	5 (10)	5 (10)	10 (20)
Increase ≥60 and QTcF≤480	0	0	0
Increase ≥60 and QTcF>480	0	0	0

DP, dihydroartemisinin-piperaquine.

If triplicate measurements are reported at a timepoint (as planned on day 1, and occasionally occurring at other visits), the data summarised is the mean of the triplicate measurements.

Day 1 pre-DP dose is considered as baseline.

Figure S1. Map of Endemicity of *Plasmodium Vivax* in Indonesia in 2010 (Elyazar, 2012)

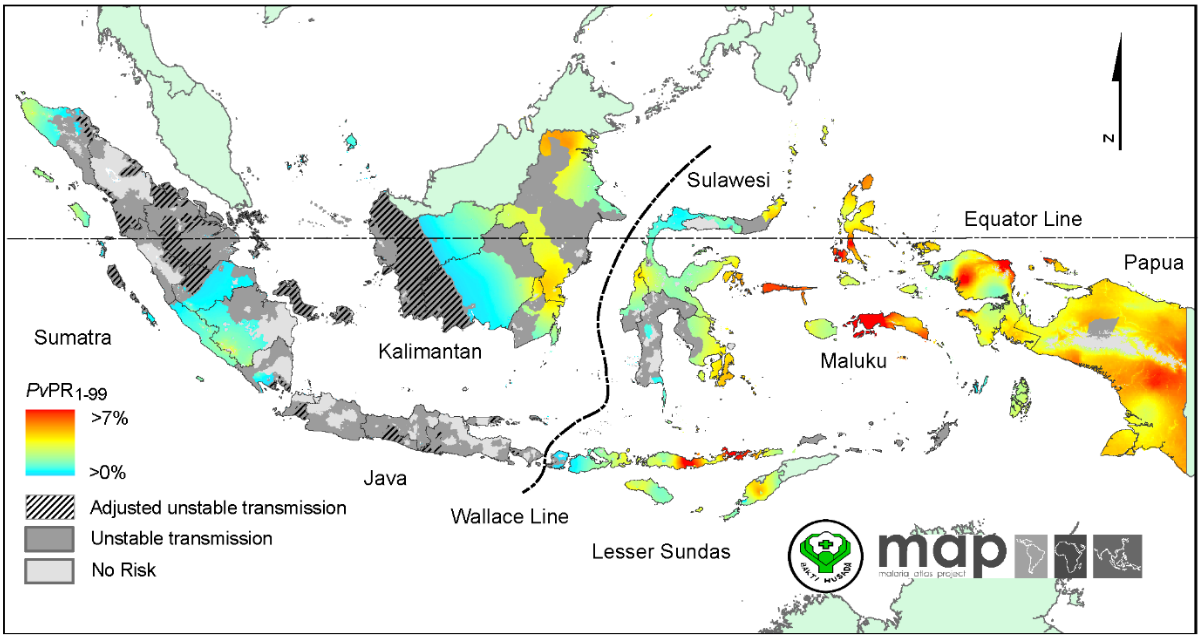

PvPR, *P. vivax* parasite rate.

Elyazar IRF, Gething PW, Patil AP, Rogayah H, Sariwati E, et al. (2012) Plasmodium vivax Malaria Endemicity in Indonesia in 2010. PLOS ONE 7(5): e37325. https://doi.org/10.1371/journal.pone.0037325 (CC-BY)

The plasmodium vivax malaria PvPR1–99 endemicity map. Model-based geostatistical point estimates of the annual mean PvPR1–99 for 2010 within the stable spatial limits of *P. vivax* malaria transmission, displayed as a continuum of light green to red from 0% to 7% (see map legend).

Figure S2. Map of Java Showing the Study Site Locations at the Two Army Bases GEOSS PROTAL European Space Agency

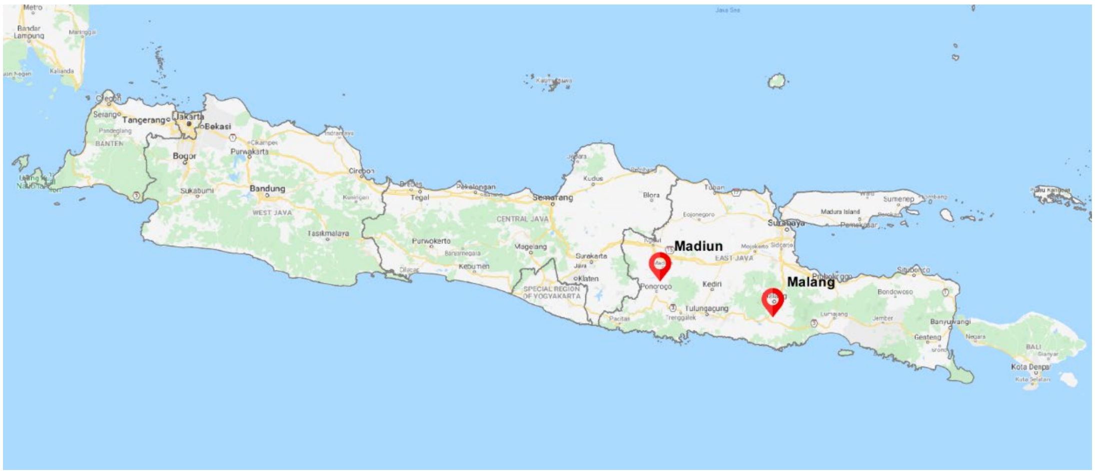

Source of map: www.geoportal.bps.go.id

Figure S3. Study 200894 Design Schematic

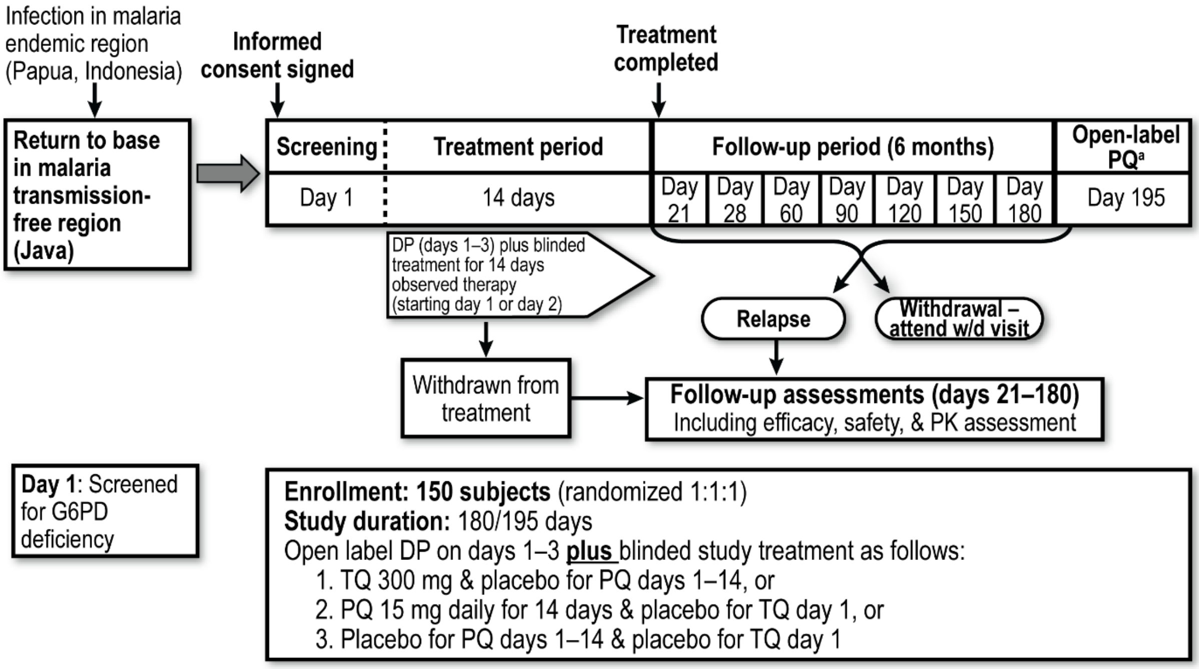

DP, dihydroartemisinin-piperaquine; PQ, primaquine; TQ, tafenoquine.

[a] Only for subjects who do not relapse.

Figure S4. Kaplan-Meier Survival Curves for Time to Relapse by Battalion (mITT Population)

Battalion 1

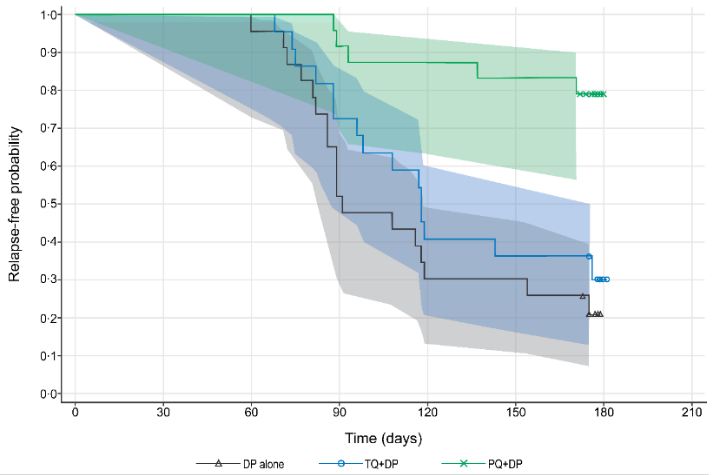

Patients at risk:								
DP alone	23	23	23	12	7	7	0	0
TQ+DP	22	22	22	16	9	8	1	0
PQ+DP	24	24	24	22	21	20	1	0

Hazard ratio: risk of relapse vs DP alone[a]	TQ+DP	PQ+DP
Estimate (95% CI)	0·700 (0·352, 1·391)	0·149 (0·055, 0·404)

Battalion 2

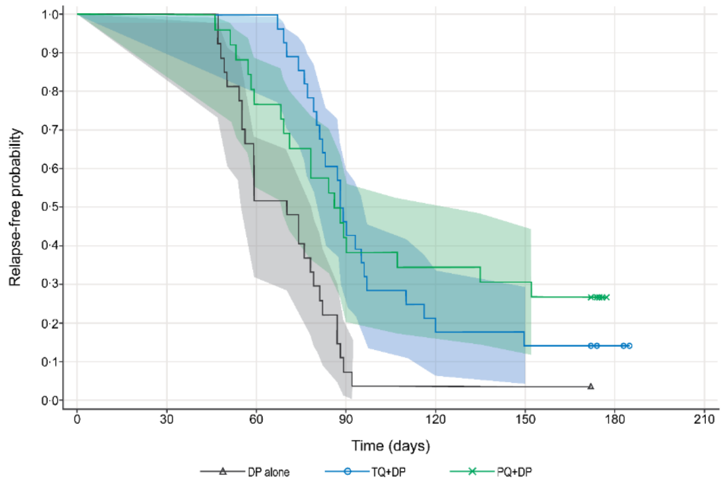

Patients at risk:								
DP alone	27	27	14	2	1	1	0	0
TQ+DP	28	28	28	13	6	5	2	0
PQ+DP	26	26	20	11	9	8	0	0

Hazard ratio: risk of relapse vs DP alone[a]	TQ+DP	PQ+DP
Estimate (95% CI)	0·376 (0·213, 0·666)	0·343 (0·185, 0·634)

CI, confidence interval; DP, dihydroartemisinin-piperaquine; mITT, modified intent to treat; PQ, primaquine; TQ, tafenoquine.

[a] Estimated from Cox's Proportional Hazards Analysis adjusting for battalion. A hazard ratio of <1 indicates a lower chance of relapse compared to the reference treatment.

Figure S5. Incidence (95% CI) of Genetically Heterologous and Homologous Relapses

Heterologous	n=38 (76%)	n=29 (58%)	n=21 (42%)
Homologous	n=6 (12%)	n=9 (18%)	n=3 (6%)

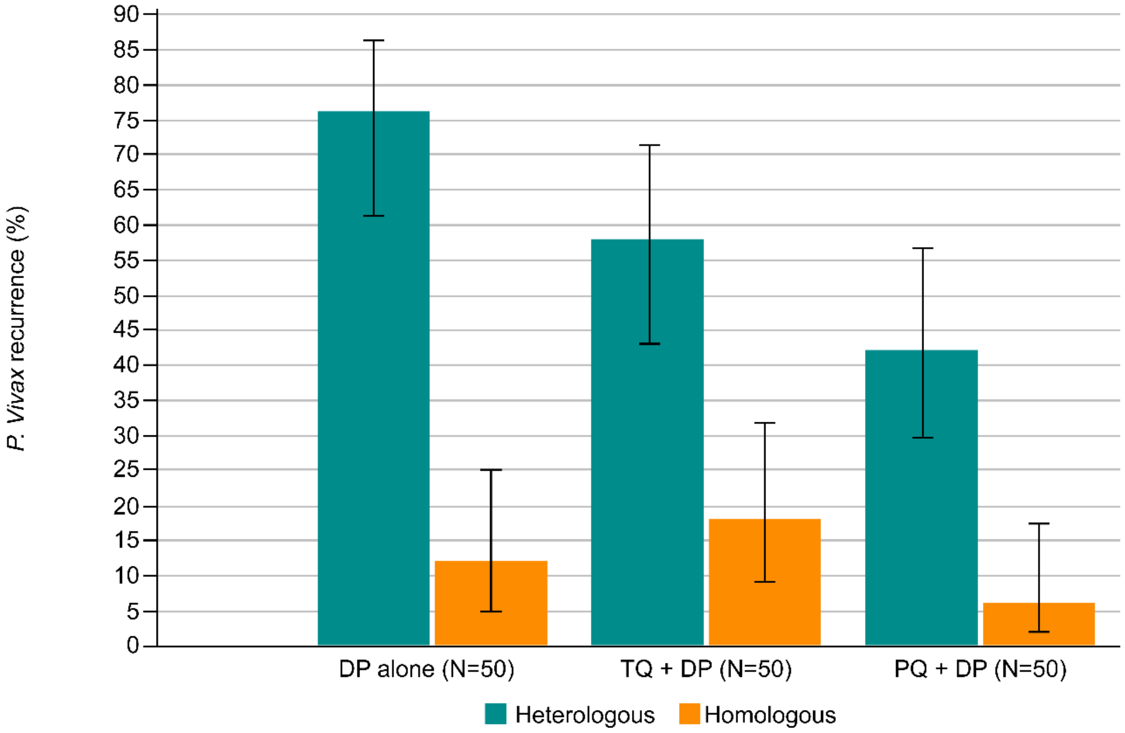

CI, confidence interval; DP, dihydroartemisinin-piperaquine; PQ, primaquine; TQ, tafenoquine.

N=number of *P. vivax* relapses.
Bars represent 95% Wilson continuity-corrected CIs.

Figure S6. Common (≥5% in any Treatment Group) AEs and Relative Risk During the Double-Blind Treatment Phase (Safety Population)

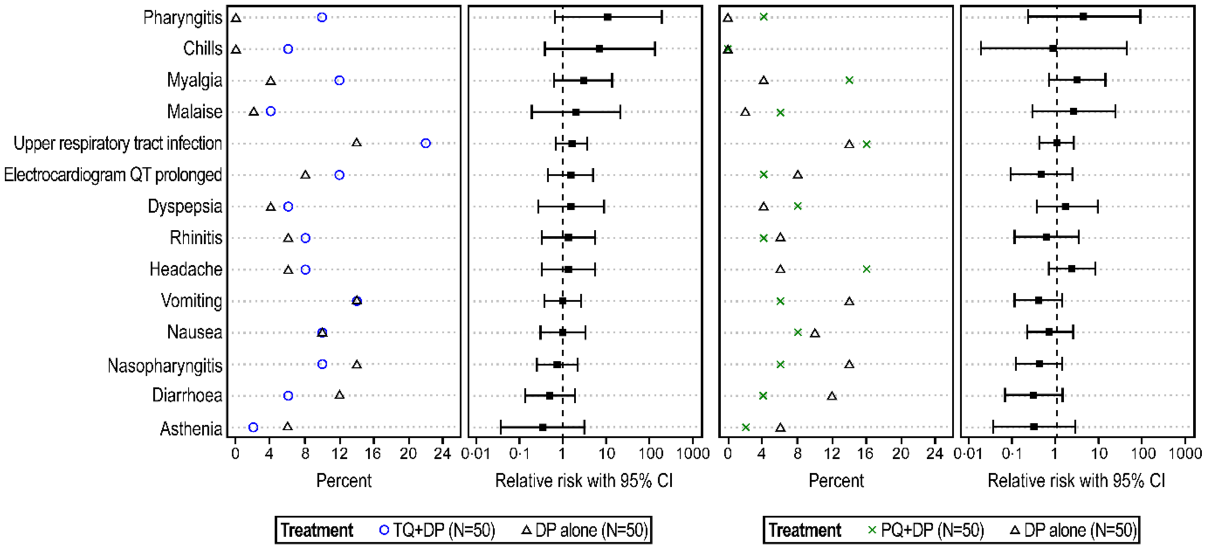

AE, adverse event; CI, confidence interval; DP, dihydroartemisinin-piperaquine; PQ, primaquine; TQ, tafenoquine.

Figure S7. Estimated Probability of Being Relapse-Free at 4 Months by Maximum Methaemoglobin A) by Treatment Group and B) Combined From the DETECTIVE Part 1, DETECTIVE Part 2, GATHER and INSPECTOR Studies (Post-Hoc Analysis)

A)

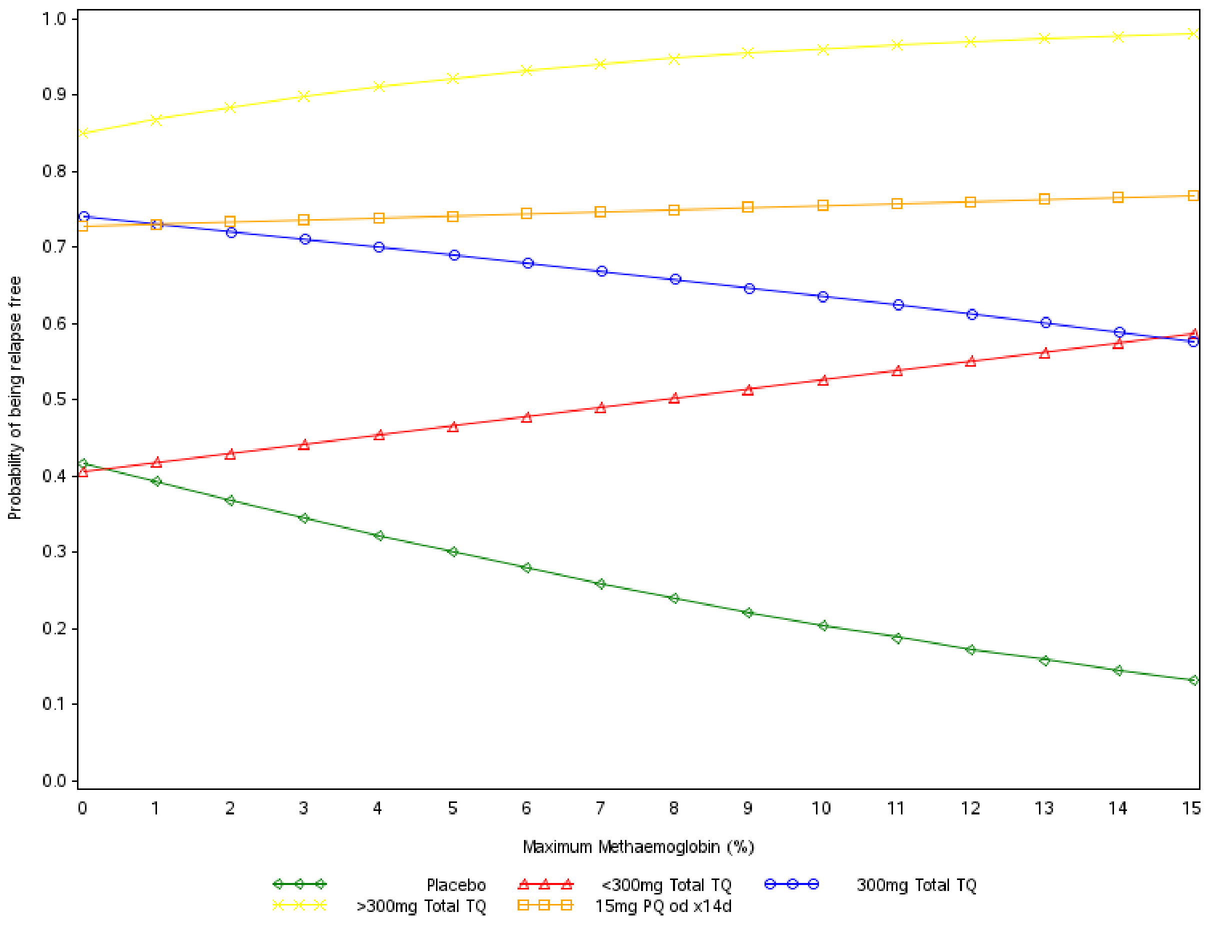

B)

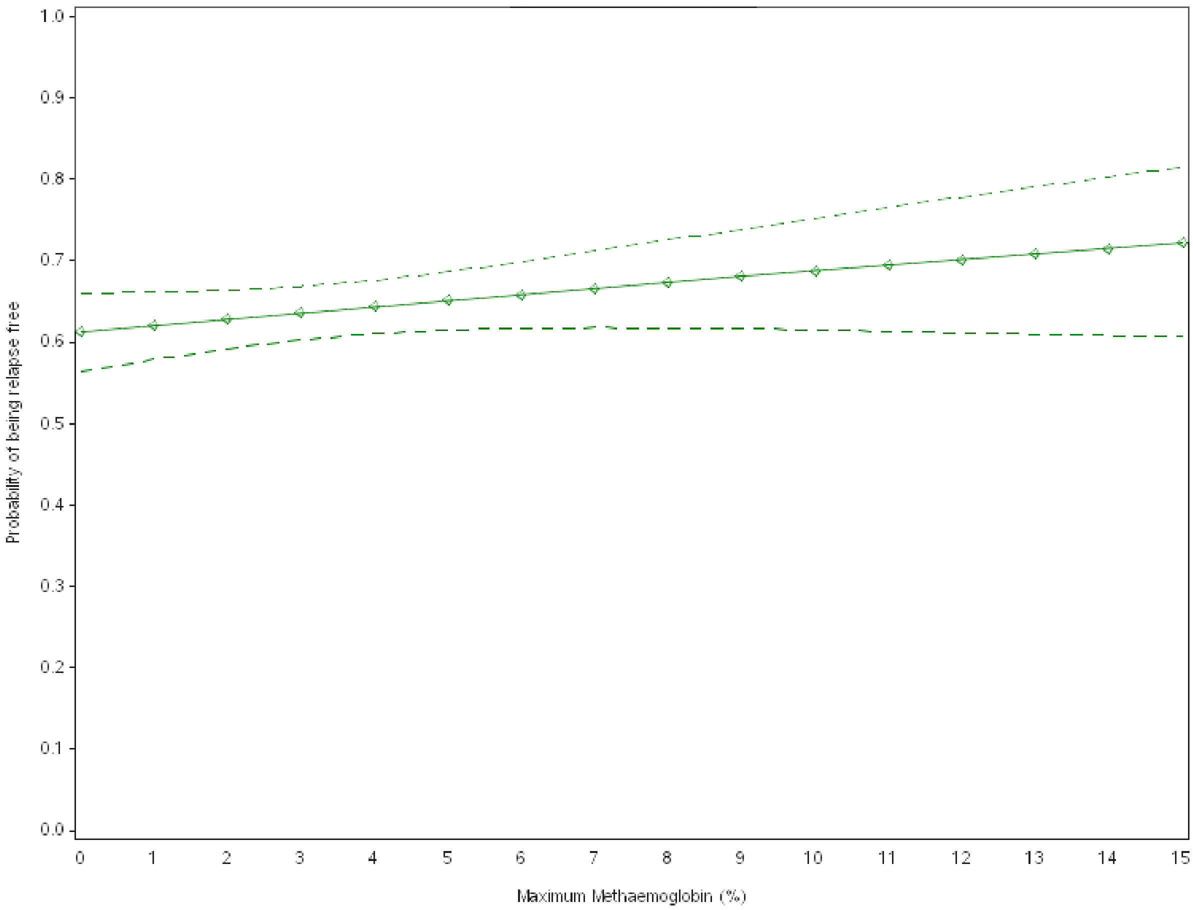

Probability of being relapse free
1.0
0.9
0.8
0.7
0.6
0.5
0.4
0.3
0.2
0.1
0.0
0
1
2
3
4
5
6
7
8
9
10
11
12
13
14
15
Maximum Methaemoglobin (%)

Supplementary References

Gaedigk A, Simon SD, Pearce RE, Bradford LD, Kennedy MJ, Leeder JS. The CYP2D6 Activity Score: Translating Genotype Information into a Qualitative Measure of Phenotype. *Clin Pharmacol Ther* 2008; **83**: 234–42.

Caudle KE, Sangkuhl K, Whirl-Carrillo M, et al. Standardizing CYP 2D6 genotype to phenotype translation: consensus recommendations from the Clinical Pharmacogenetics Implementation Consortium and Dutch Pharmacogenetics Working Group. *Clin Transl Sci* 2020; **13**(1): 116–24.

Elyazar IRF, Gething PW, Patil AP, et al. Plasmodium vivax malaria endemicity in Indonesia in 2010. *PloS One* 2012; **7**(5): e37325-e37325.
